# Supplementary material for: Cytosolic Ptbp2 modulates axon growth in motoneurons through axonal localization and translation of Hnrnpr
Source: Nat Commun. 2023 Jul 12;14:4158. doi: 10.1038/s41467-023-39787-6 (PMC10338680; doi:10.1038/s41467-023-39787-6)
Supplement: Supplementary file 1 — Supplementary Information [file 41467_2023_39787_MOESM1_ESM.pdf]

Supplementary Information for

Cytosolic Ptbp2 modulates axon growth in motoneurons through axonal localization and translation of *Hnrnpr*

Saeede Salehi, Abdolhossein Zare, Gianluca Prezza, Jakob Bader, Cornelius Schneider, Utz Fischer, Felix Meissner, Matthias Mann, Michael Briese, Michael Sendtner

This PDF file includes:

Supplementary Figures 1 to 5

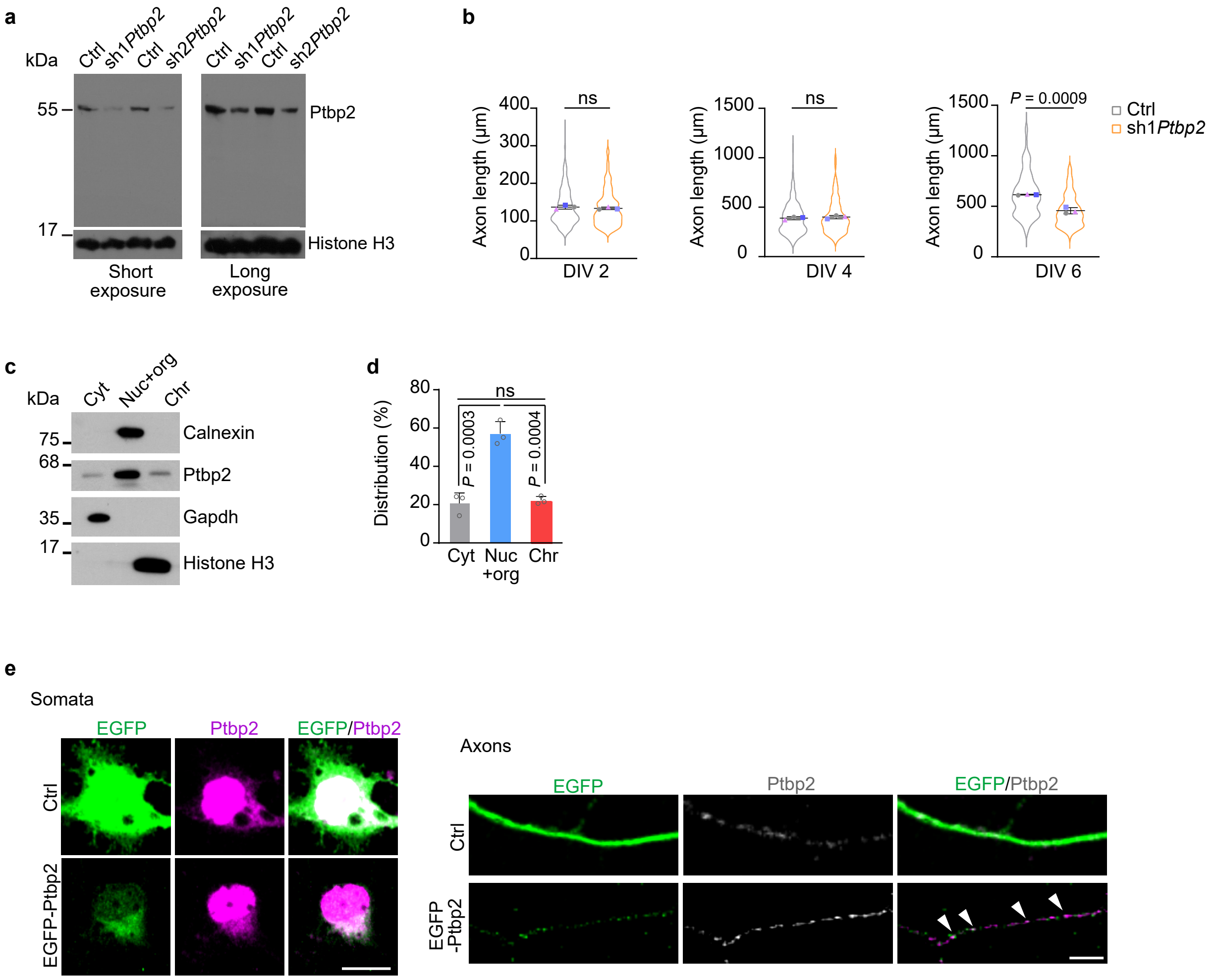

Supplementary Figure 1. Ptbp2 is present in the cytosolic fraction and regulates axon growth.

a, Immunoblot of Ptbp2 in control and Ptbp2-depleted motoneurons at DIV 6. Histone H3 was used as a loading control. **b**, SuperPlots of axon lengths of control and Ptbp2-depleted motoneurons at DIV 2, 4 and 6. Unpaired two-tailed Student's t-test. Data are mean ± s.d. of *n*=3 biological replicates. **c**, Immunoblot analysis of subcellular fractions of NSC-34 cells. Cyt, cytosol; Nuc+org, nuclear soluble proteins and organelles; Chr, chromatin-associated proteins. the immunoblots are representative of three biological replicates. **d**, Quantification of Western blot signals in (**c**). One-way ANOVA with Tukey's multiple comparisons test. Data are mean ± s.d. of *n*=3 biological replicates. **e**, Immunostaining for EGFP and Ptbp2 of motoneurons transduced with a control lentivirueses, or a lentivirus expressing an EGFP-Ptbp2 fusion protein. The images are representative of three biological replicates. Source data are provided as a Source Data file.

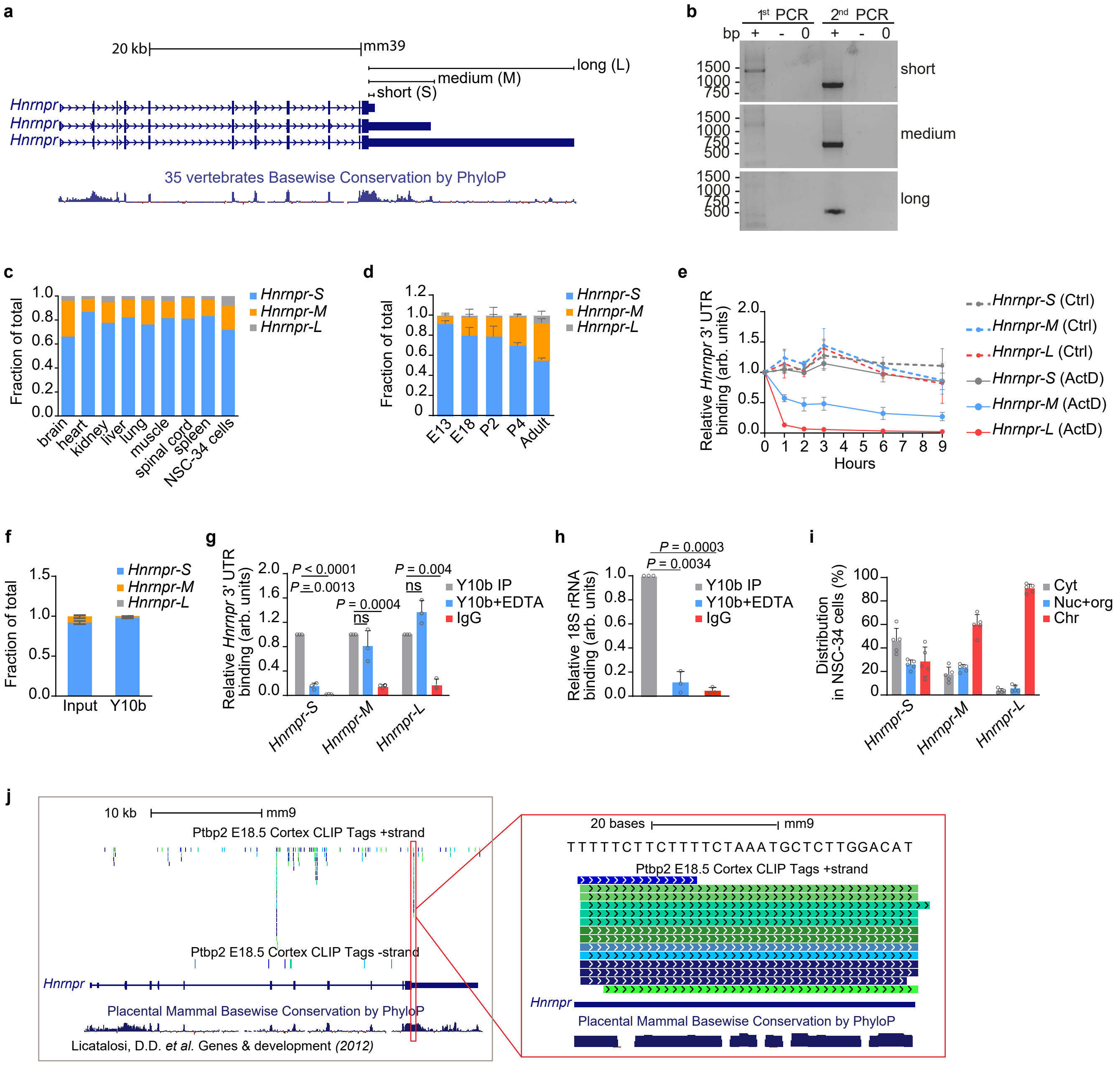

Supplementary Figure 2. Alternative polyadenylation analysis of *Hnrnpr*.

a, UCSC genome browser view of the mouse *Hnrnpr* locus. Blue boxes indicate exons, lines are introns. The lower trace represents conservation across 35 vertebrates. Positioning of the short (S), medium (M) and long (L) 3' UTR is indicated. **b**, Detection of *Hnrnpr* 3' UTRs in mouse brain by 3' RACE. The 2nd PCR was performed with a nested forward primer using the product of 1st PCR as a template. +, RT reaction as template; -, no reverse transcriptase control; 0, no template PCR control. The gele are representative of at least two biological replicates. **c**, *Hnrnpr* 3' UTR expression across mouse tissues and NSC-34 cells. Each isoform is shown as a fraction of the total UTR isoforms. **d**, *Hnrnpr* 3' UTR expression in the mouse spinal cord at various developmental stages. Data are mean ± s.d. of $n = 3$ biological replicates. **e**, Stability analysis of *Hnrnpr* 3' UTR isoforms measured by qPCR at different time points following Actinomycin D treatment of NSC-34 cells. ActD, RNA from Actinomycin D-treated cells; Ctrl, RNA from vehicle-treated cells (0.01% DMSO final concentration). Data are mean ± s.d. of $n = 3$ biological replicates. **f**, Distribution of the *Hnrnpr* 3' UTR isoforms in the input and following ribosome pulldown using the Y10b antibody. Data are mean ± s.d. of $n = 3$ biological replicates. **g**, RNA immunoprecipitation of the *Hnrnpr* 3' UTR isoforms following purification with the Y10b antibody in the absence or presence of EDTA, or with IgG control. Two-way ANOVA. Data are mean ± s.d. of $n = 3$ biological replicates. **h**, RNA immunoprecipitation of 18S rRNA following purification with the Y10b antibody in the absence or presence of EDTA, or with IgG control. One-way ANOVA. Data are mean ± s.d. of $n = 3$ biological replicates. **i**, Distribution of *Hnrnpr* 3' UTRs in different fractions of NSC-34 cells. Data are mean ± s.d. of $n = 5$ biological replicates. **j**, UCSC genome browser track of Ptbp2 CLIP-Seq reads along the Hnrnpr locus. Magnified area indicates peak of Ptbp2 CLIP-Seq sites in the *Hnrnpr* 3' UTR. Source data are provided as a Source Data file.

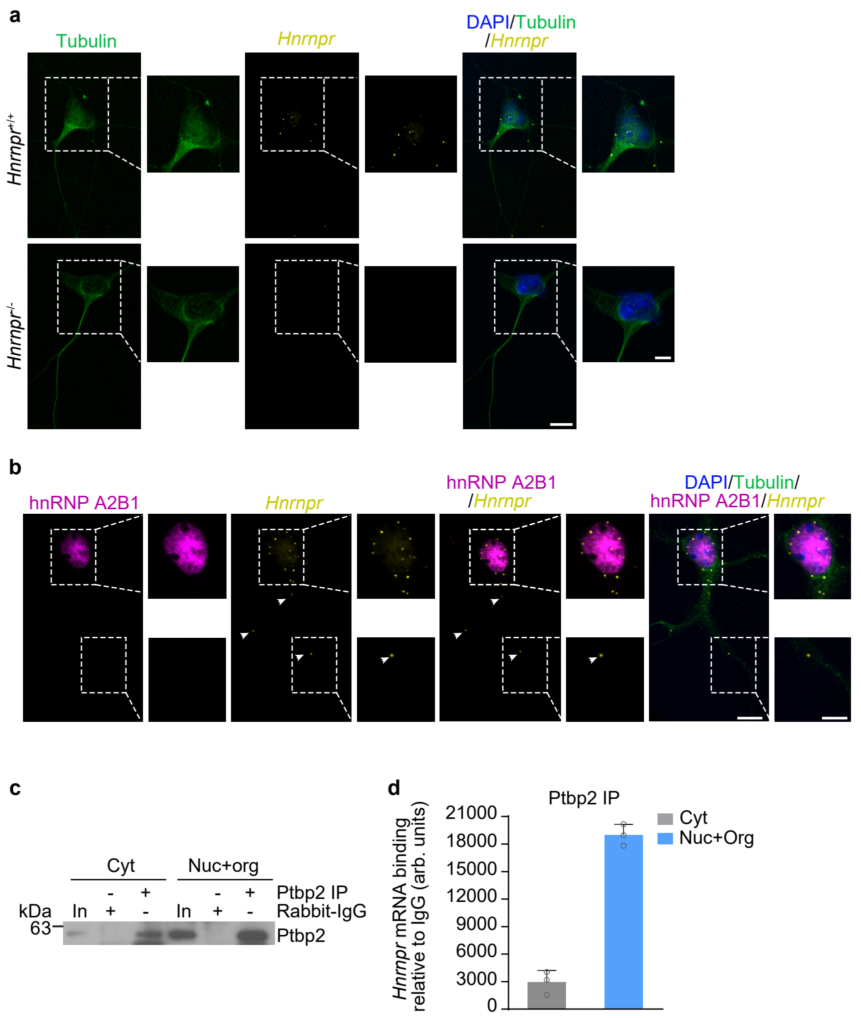

Supplementary Figure 3. Interaction of Ptbp2 with the *Hnrnpr* 3' UTR in the cytosolic fraction of motoneurons.

a, Representative images of *Hnrnpr* FISH in cultured *Hnrnpr*$^{+/+}$ and *Hnrnpr*$^{-/-}$ motoneurons at DIV 6. An antibody against Tubulin was used for visualization of motoneuron morphology. Scale bars, 10 µm and 5 µm (magnified areas). The images are representative of two biological replicates. **b**, Representative images showing hnRNP A2B1 immunofluorescence and *Hnrnpr* FISH in cultured motoneurons at DIV 6. An antibody against Tubulin was used for visualization of motoneuron morphology. Arrowheads indicate localization of *Hnrnpr* in axon. Scale bars, 10 µm and 5 µm (magnified areas). The images are representative of three biological replicates. **c**, Immunoblot analysis of Ptbp2 immunoprecipitation from the cytosolic (Cyt) fraction and the fraction containing nuclear soluble proteins and organelles (Nuc+org) of motoneurons. **d**, qPCR analysis of *Hnrnpr* co-precipitated by anti-Ptbp2 from motoneuron fractions. Data are mean ± s.d. of $n = 3$ biological replicates. Source data are provided as a Source Data file.

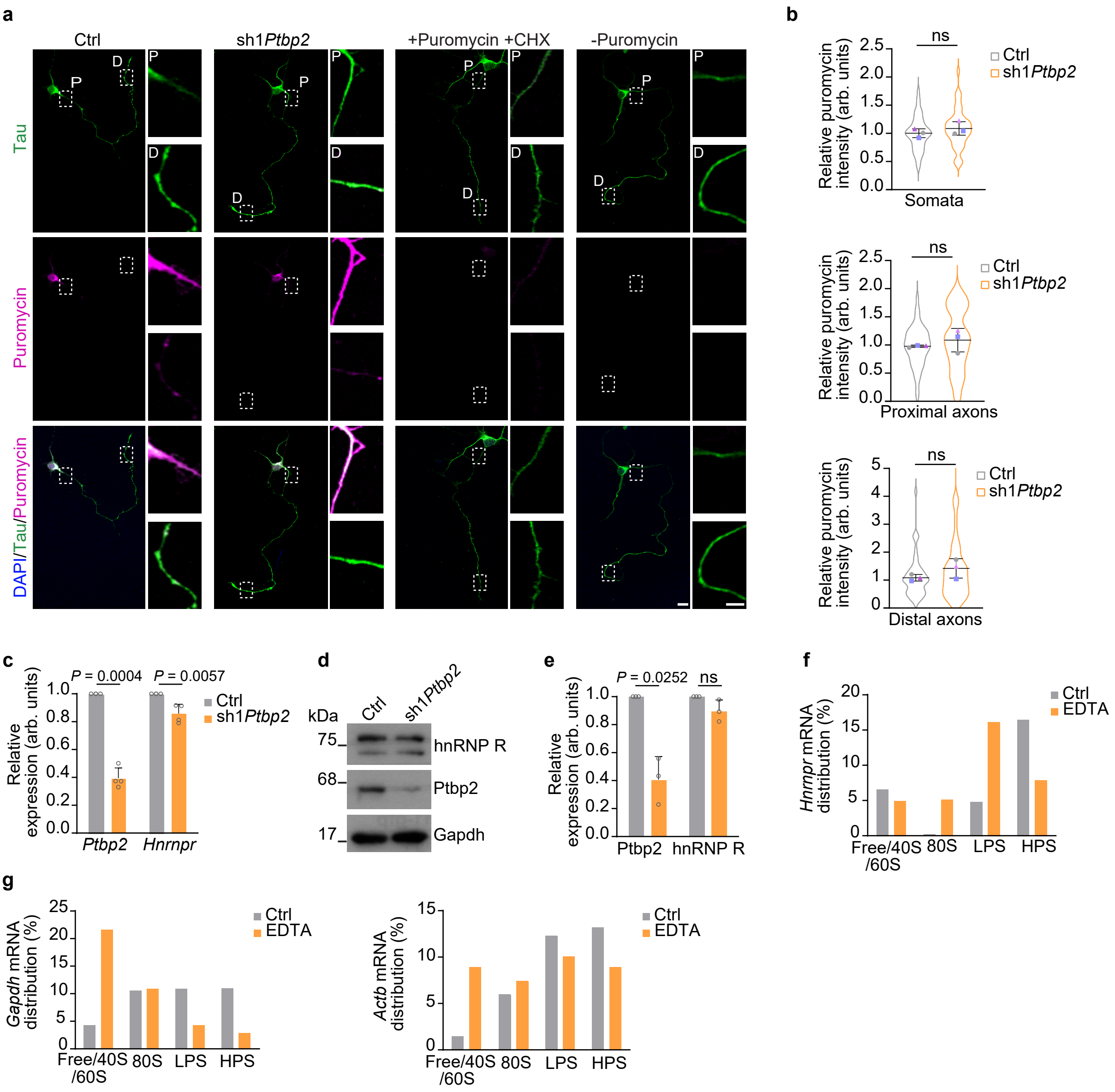

Supplementary Figure 4. Ptbp2 regulates hnRNP R translation.

a, Puromycin labeling and immunostaining in control and Ptbp2-depleted motoneurons at DIV 6. Proximal (P) and distal (D) regions of axon are marked by boxes. Scale bar, 10 µm and 5 µm (magnified areas). **b**, SuperPlots of puromycin immunosignals. Unpaired two-tailed Student's t-test. Data are mean ± s.d. of *n* = 3 biological replicates. **c**, Quantification of *Hnrnpr* and *Ptbp2* mRNA levels by qPCR in NCS-34 cells 72 h after transduction with a *Ptbp2* knockdown (sh1*Ptbp2*) or control (Ctrl) lentivirus. *Gapdh* was used for normalization. Two-tailed one-sample t-test. Data are mean ± s.d. of *n* = 3 biological replicates. **d**, Immunoblot analysis of Ptbp2 and hnRNP R protein levels from control and Ptbp2-depleted NSC-34 cells. Gapdh was used as a loading control. **e**, Quantification of the blots in (**d**). Two-tailed one-sample t-test. Data are mean ± s.d. of *n* = 3 biological replicates. **f**,**g**, Relative distribution of *Hnrnpr* (**f**), *Gapdh* and *Actb* (**g**) in motoneuron sucrose gradient fractions in the presence and absence of EDTA. LPS, light polysome fraction; HPS, heavy polysome fraction. Source data are provided as a Source Data file.

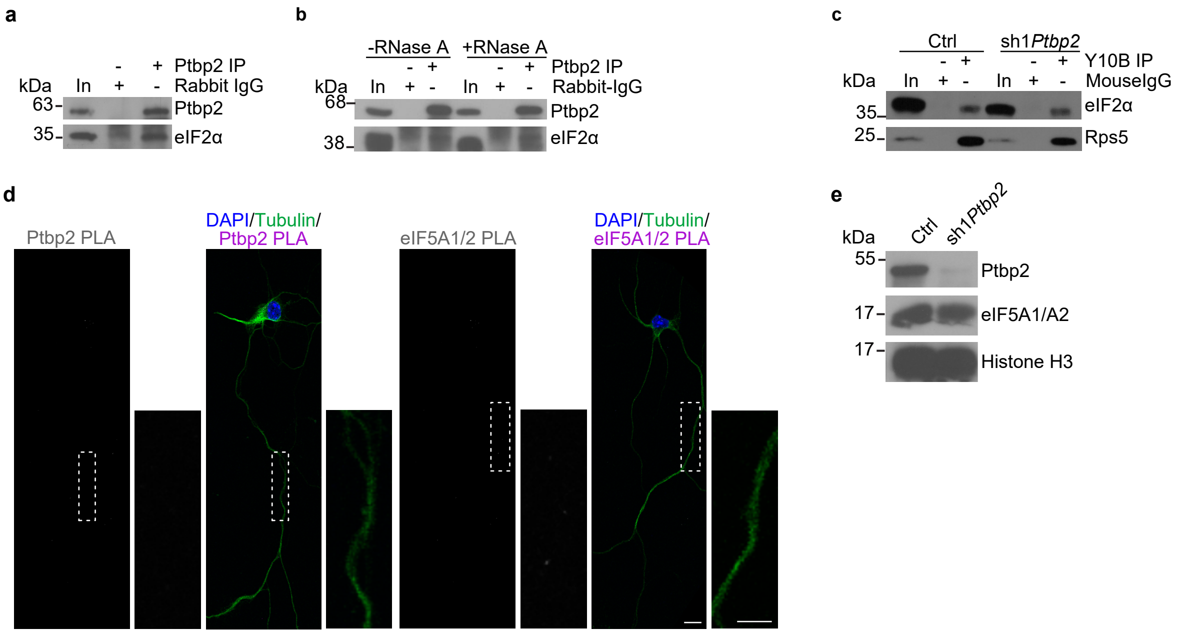

Supplementary Figure 5. Ptbp2-mediated association of *Hnrnpr* with ribosomes involves eIF5A2.

a, Co-immunoprecipitation of eIF2α by anti-Ptbp2 from motoneurons. **b**, Immunoblot analysis of eIF2α co-immunprecipitated by anti-Ptbp2 from motoneuron lysate pre-treated with RNase A as indicated. **c**, Co-immunoprecipitation of eIF2α by Y10b from control and Ptbp2-depleted motoneurons. **d**, Representative images of PLA signal in motoneurons at DIV 6 with either Ptbp2 or eIF5A1/2 antibody alone as a negative control. Motoneuron morphology was visualized by Tubulin immunofluorescence. Scale bars, 10 µm and 5 µm (magnified areas). **e**, Immunoblot analysis of Ptbp2 and eIF5A1/2 protein levels in control and Ptbp2-depleted motoneurons. Histone H3 was used as loading control. All experiments were repeated at least three times independently with similar results. Source data are provided as a Source Data file.
