# Supplementary material for: Cytosolic Ptbp2 modulates axon growth in motoneurons through axonal localization and translation of Hnrnpr
Source: Nat Commun. 2023 Jul 12;14:4158. doi: 10.1038/s41467-023-39787-6 (PMC10338680; doi:10.1038/s41467-023-39787-6)
Supplement: Supplementary file 3 — Description of Additional Supplementary Files [file 41467_2023_39787_MOESM3_ESM.pdf]

**Description of Additional Supplementary Files**

File Name: Supplementary Data 1

Description: List of proteins identified by mass spectrometry following pulldown with the Hnrnpr 3' UTR.

File Name: Supplementary Data 2

Description: Primer sequences for RT-qPCR.

File Name: Supplementary Data 3

Description: List of primary and secondary antibodies.
