# Supplementary figures and images for: Cytosolic Ptbp2 modulates axon growth in motoneurons through axonal localization and translation of Hnrnpr
Source: Nat Commun. 2023 Jul 12;14:4158. doi: 10.1038/s41467-023-39787-6 (PMC10338680; doi:10.1038/s41467-023-39787-6)

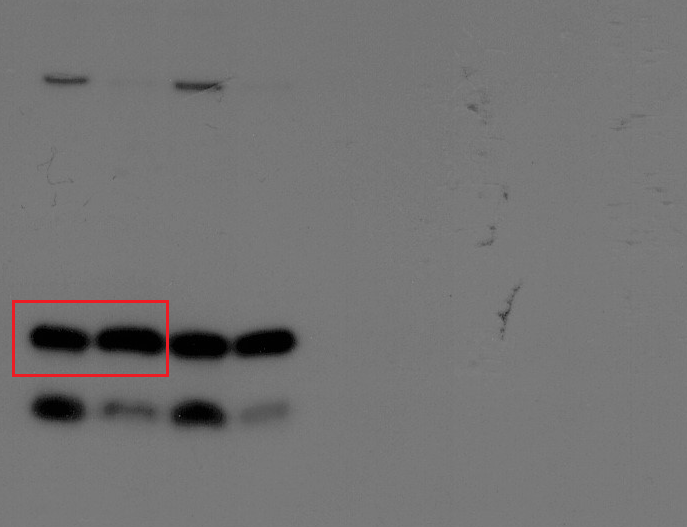

Supplement: Supplementary file 8 — Source Data [file 41467_2023_39787_MOESM8_ESM.zip › gels_blots/Figure 1/Fig. 1b-Histone H3(sh2Ptbp2).tif]

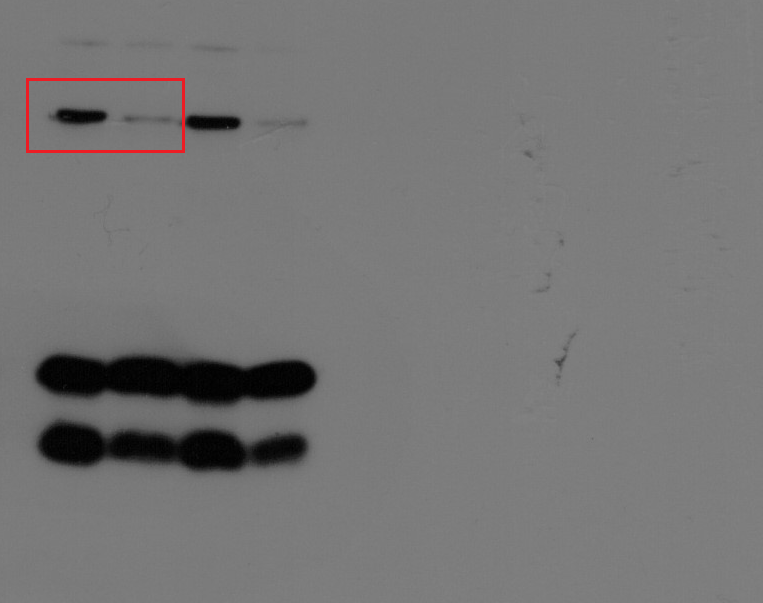

Supplement: Supplementary file 8 — Source Data [file 41467_2023_39787_MOESM8_ESM.zip › gels_blots/Figure 1/Fig. 1b-Ptbp2(sh2Ptbp2).tif]

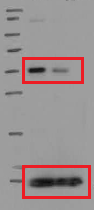

Supplement: Supplementary file 8 — Source Data [file 41467_2023_39787_MOESM8_ESM.zip › gels_blots/Figure 1/Fig. 1b-Ptbp2-Histone H3(sh1Ptbp2).tif]

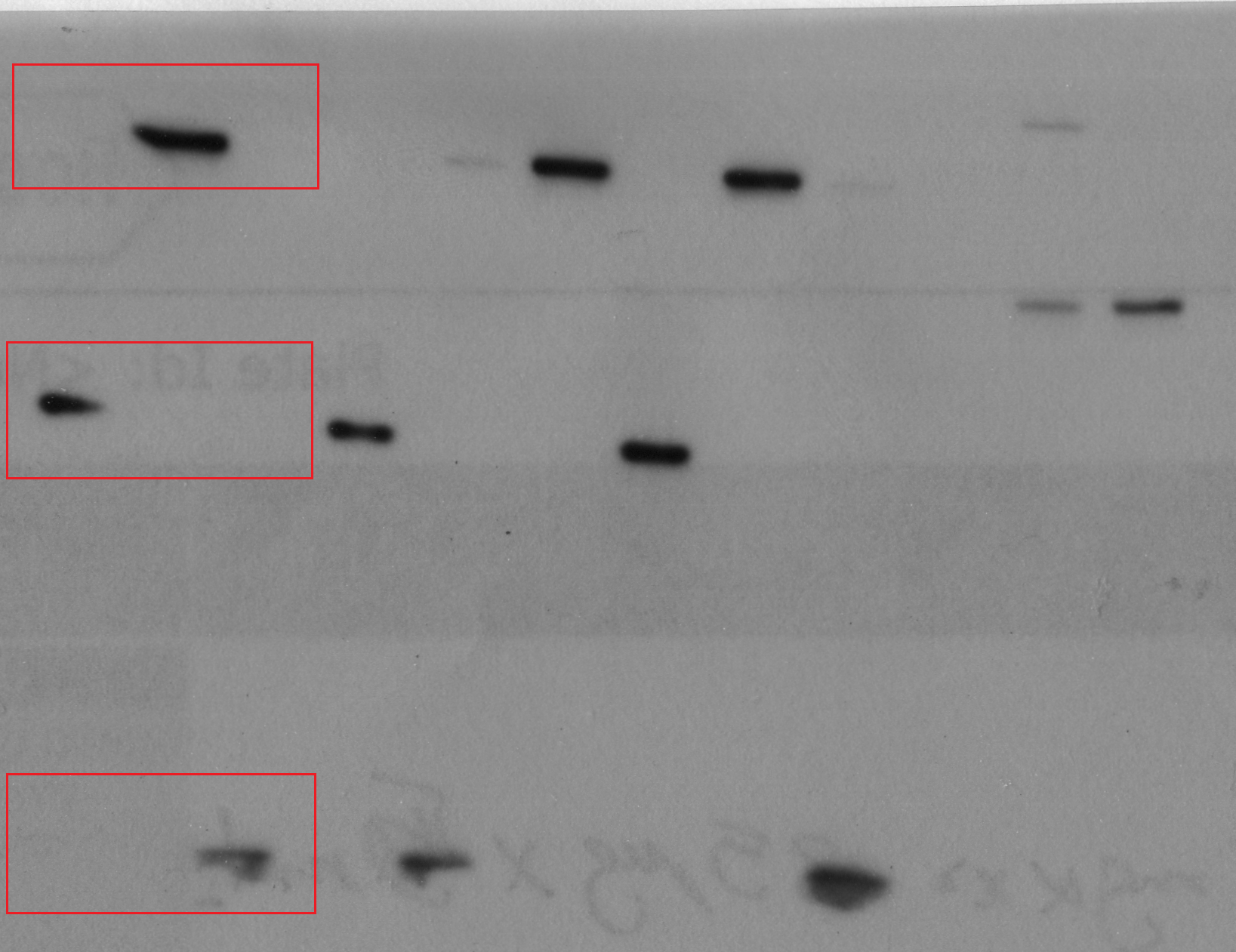

Supplement: Supplementary file 8 — Source Data [file 41467_2023_39787_MOESM8_ESM.zip › gels_blots/Figure 2/Fig. 2a-Calnexin-Gapdh-Histone H3.tif]

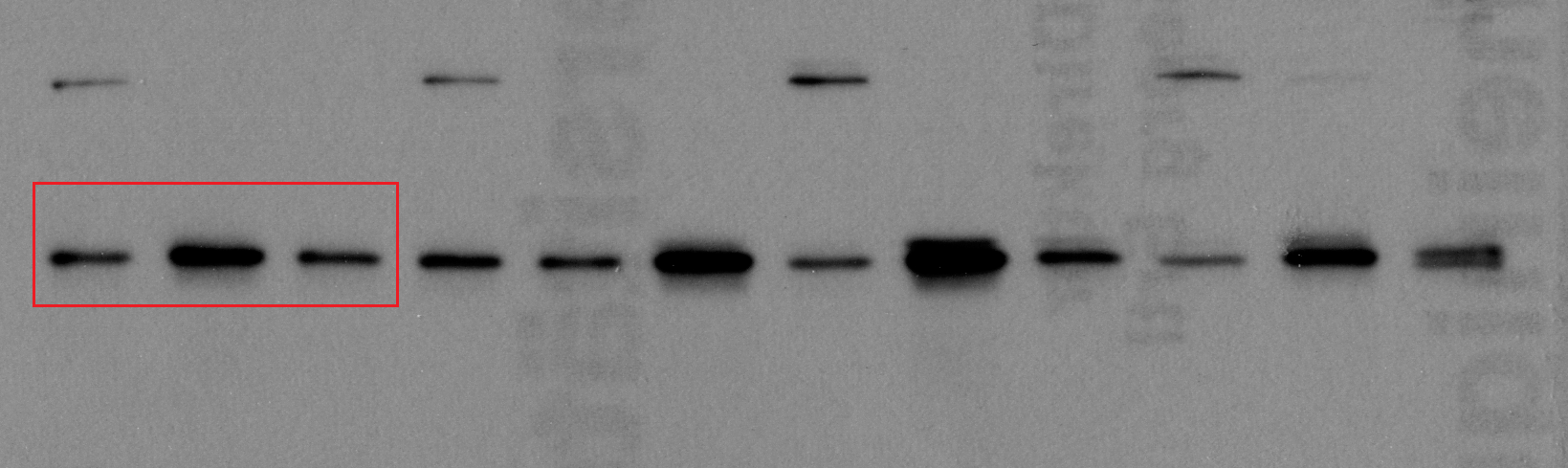

Supplement: Supplementary file 8 — Source Data [file 41467_2023_39787_MOESM8_ESM.zip › gels_blots/Figure 2/Fig. 2a-Ptbp2.tif]

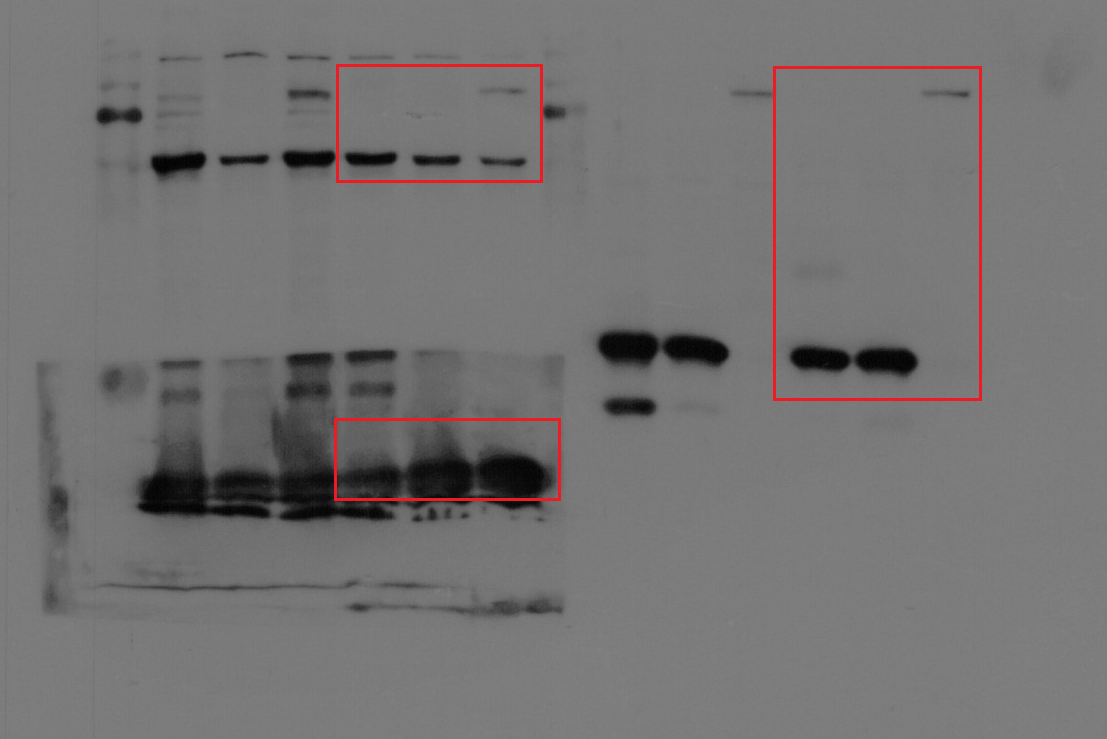

Supplement: Supplementary file 8 — Source Data [file 41467_2023_39787_MOESM8_ESM.zip › gels_blots/Figure 2/Fig. 2e-Ptbp2-Histone H3-EGFP.tif]

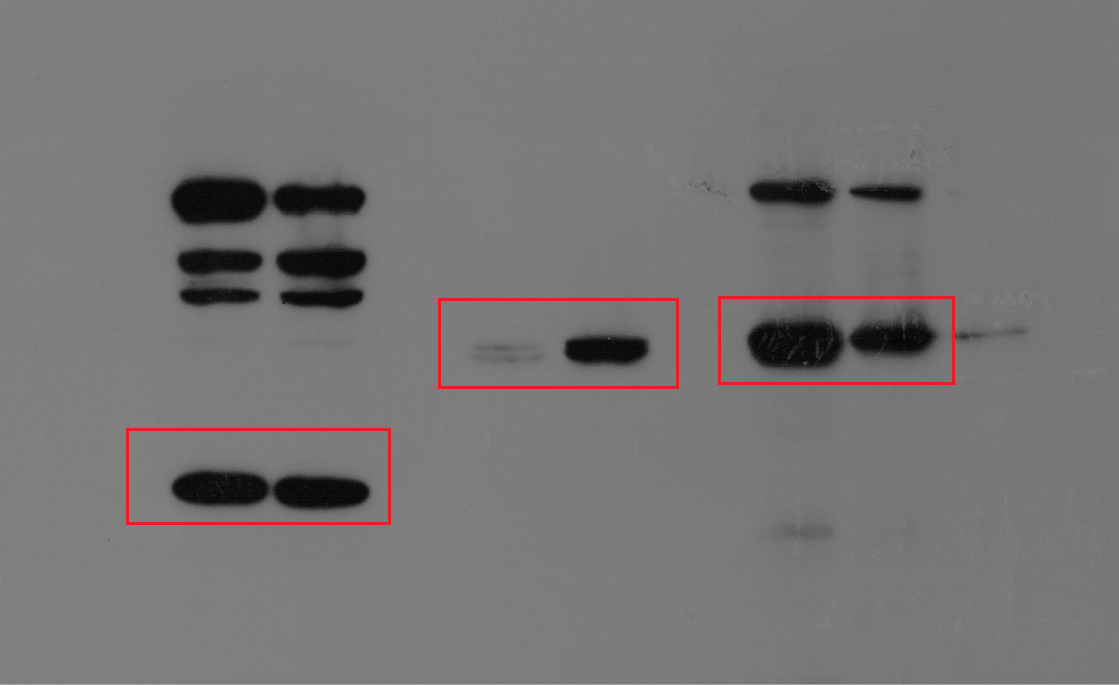

Supplement: Supplementary file 8 — Source Data [file 41467_2023_39787_MOESM8_ESM.zip › gels_blots/Figure 3/Fig. 3a-Ptbp2-Ptbp1-Gapdh.tif]

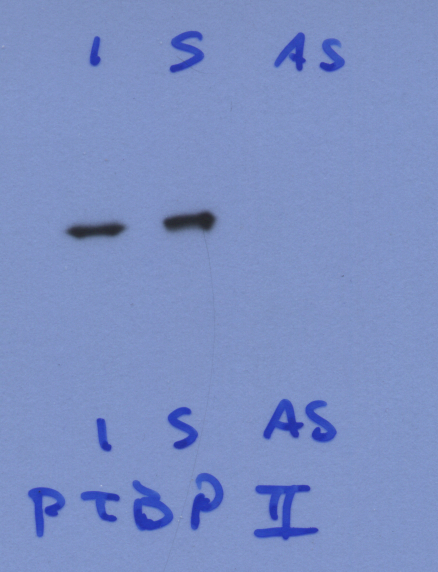

Supplement: Supplementary file 8 — Source Data [file 41467_2023_39787_MOESM8_ESM.zip › gels_blots/Figure 3/Fig. 3d-Ptbp2.tif]

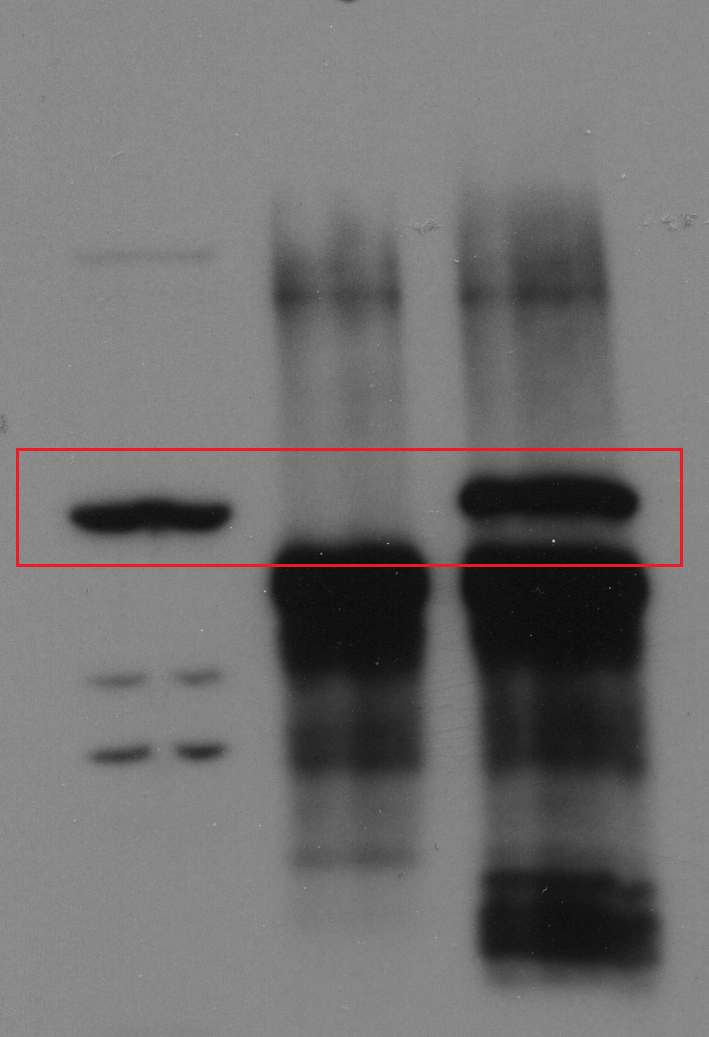

Supplement: Supplementary file 8 — Source Data [file 41467_2023_39787_MOESM8_ESM.zip › gels_blots/Figure 3/Fig. 3f-Ptbp2.tif]

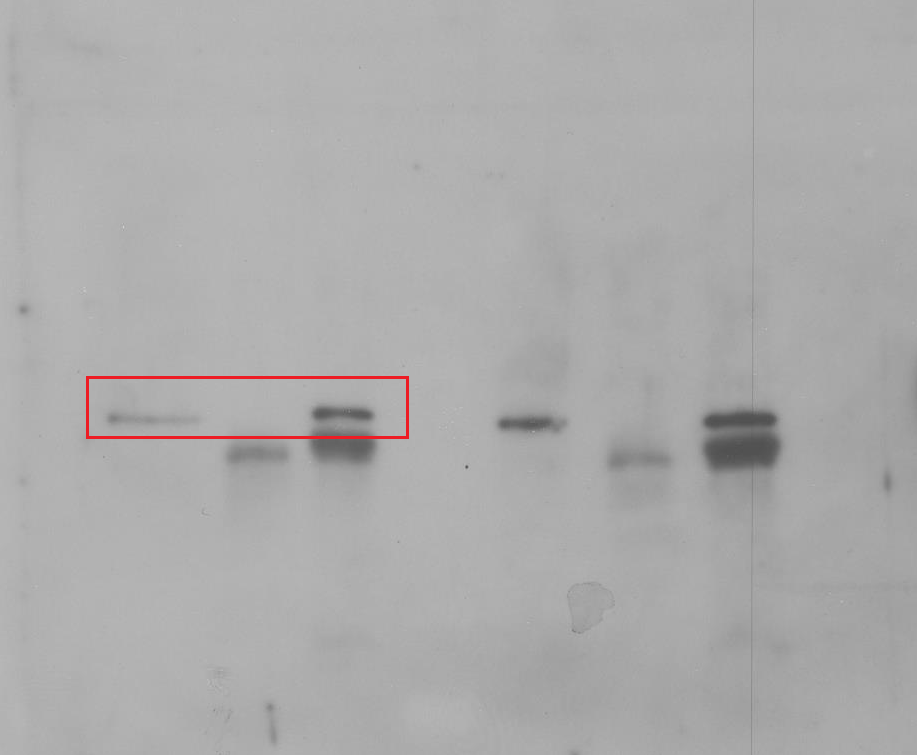

Supplement: Supplementary file 8 — Source Data [file 41467_2023_39787_MOESM8_ESM.zip › gels_blots/Figure 3/Fig. 3h-Ptbp2.tif]

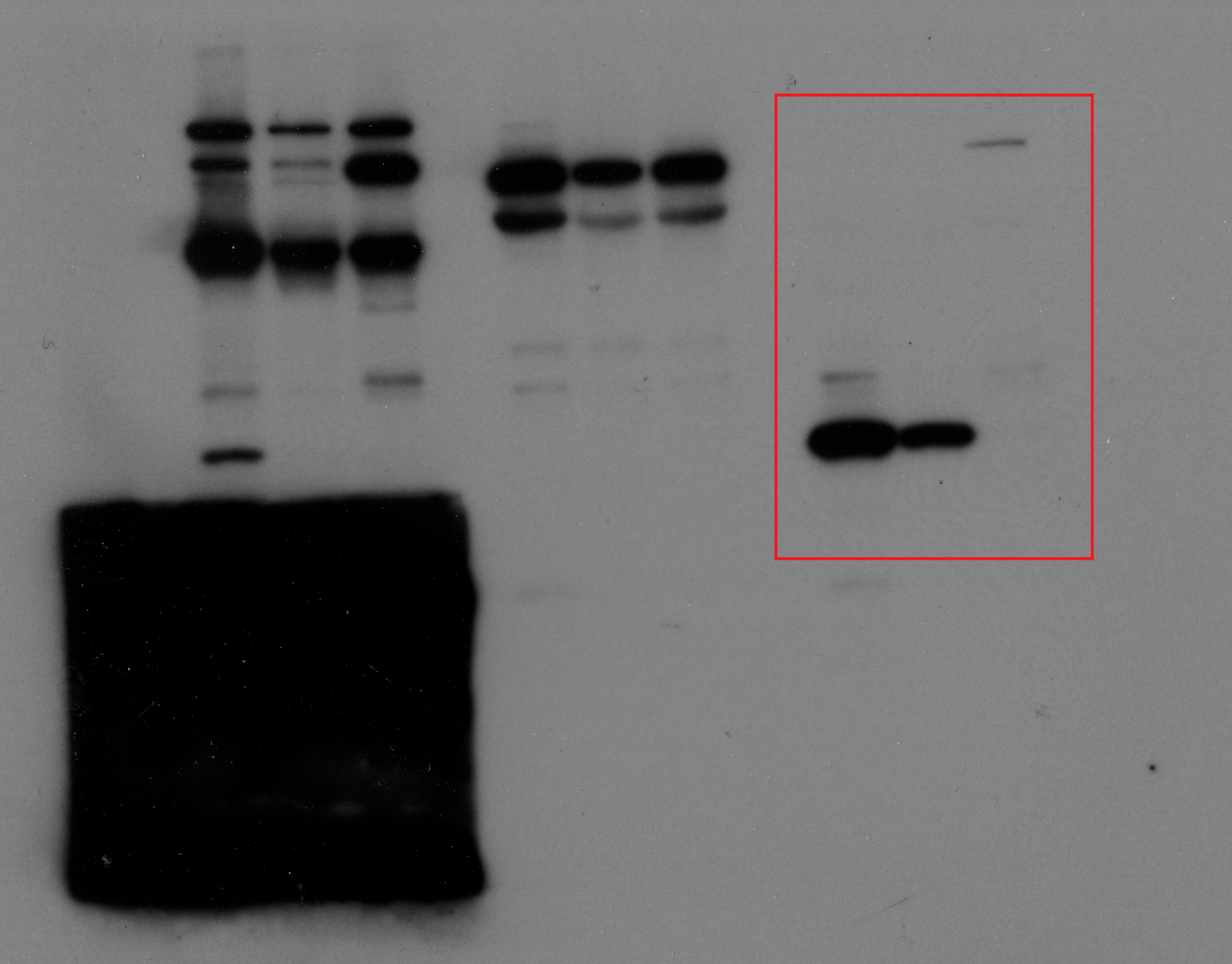

Supplement: Supplementary file 8 — Source Data [file 41467_2023_39787_MOESM8_ESM.zip › gels_blots/Figure 5/Fig. 5h-EGFP.tif]

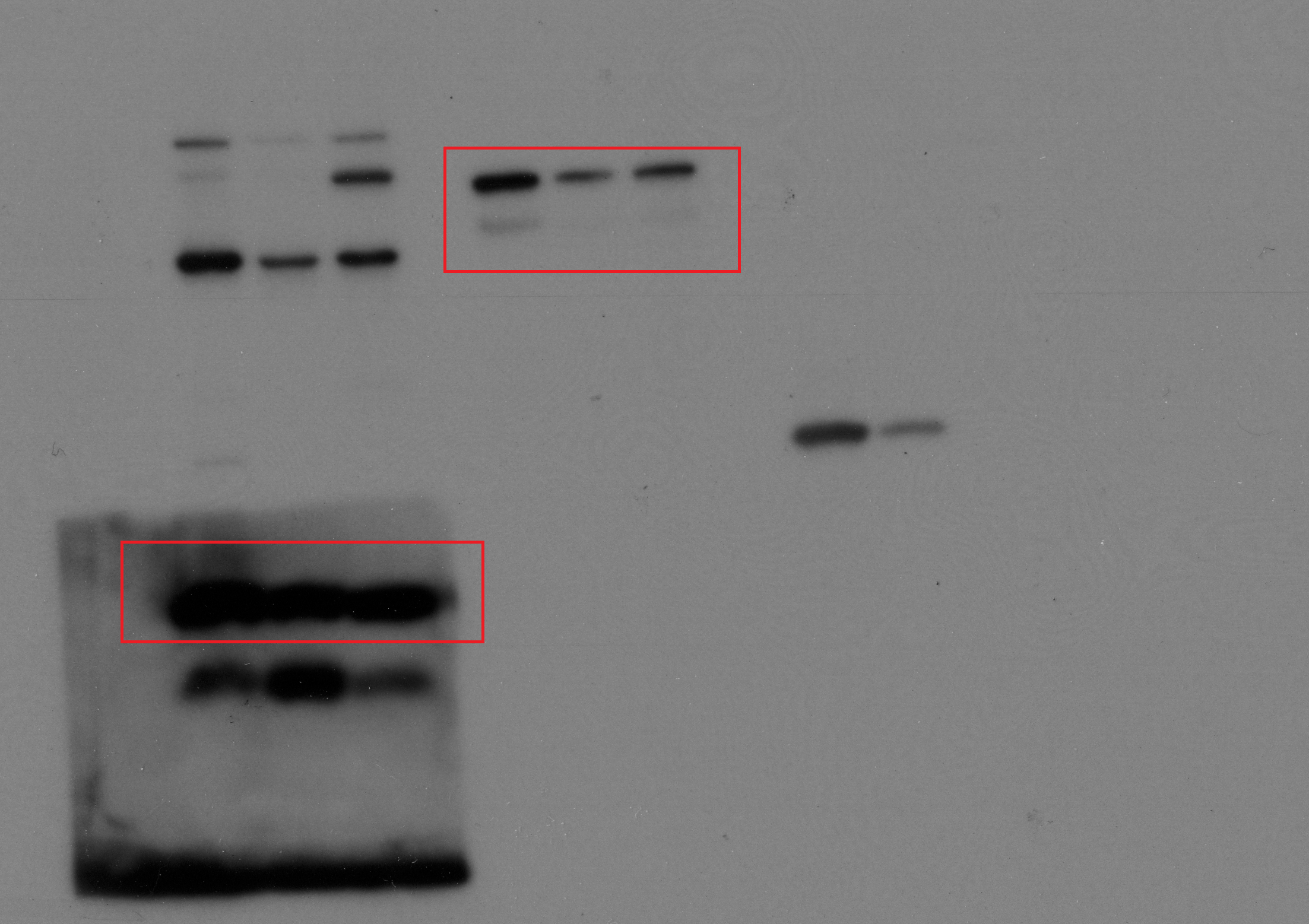

Supplement: Supplementary file 8 — Source Data [file 41467_2023_39787_MOESM8_ESM.zip › gels_blots/Figure 5/Fig. 5h-Histon h3-hnRNP R.tif]

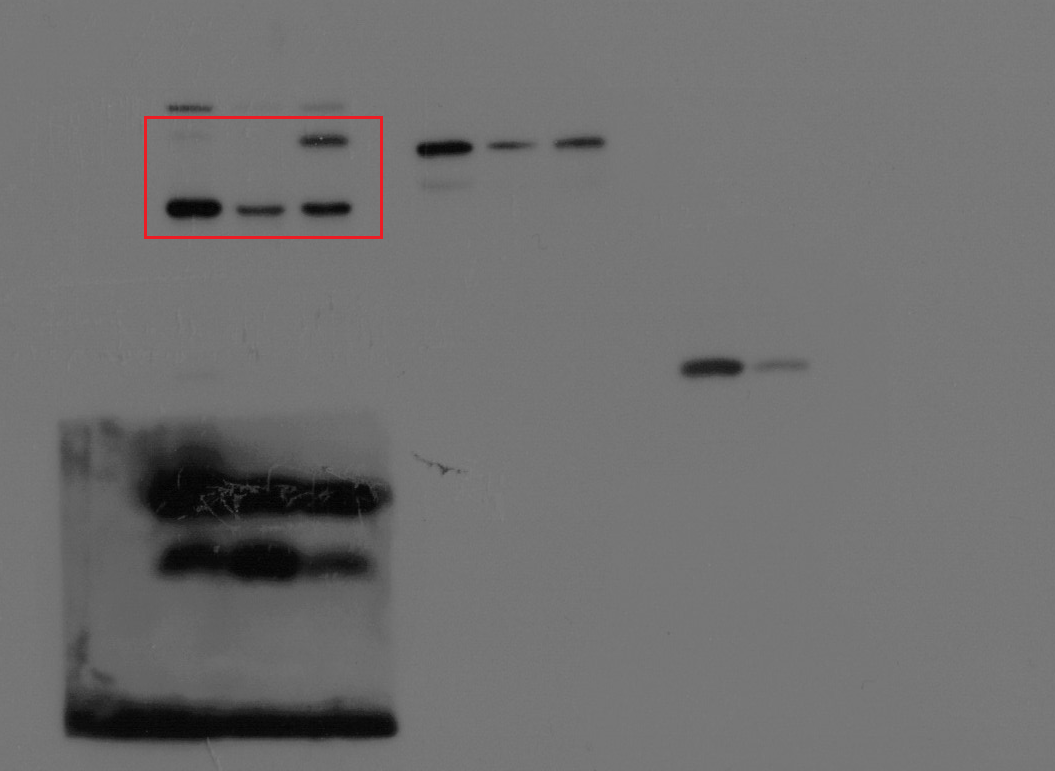

Supplement: Supplementary file 8 — Source Data [file 41467_2023_39787_MOESM8_ESM.zip › gels_blots/Figure 5/Fig. 5h-Ptbp2.tif]

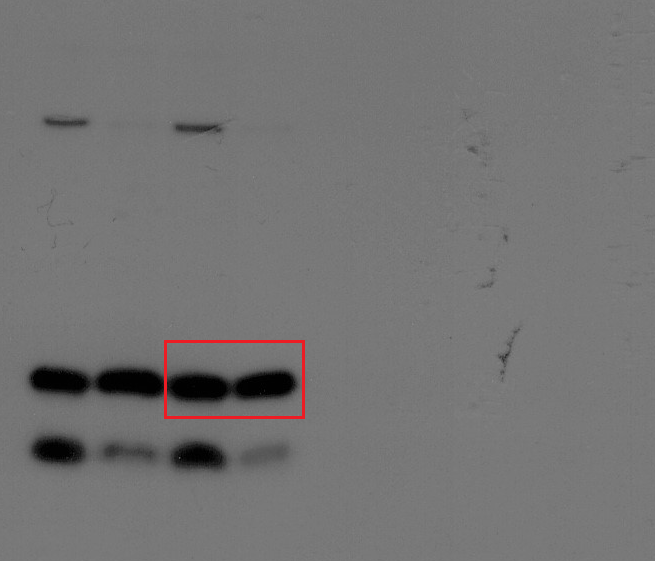

Supplement: Supplementary file 8 — Source Data [file 41467_2023_39787_MOESM8_ESM.zip › gels_blots/Figure 5/Fig. 5j-Histone H3.tif]

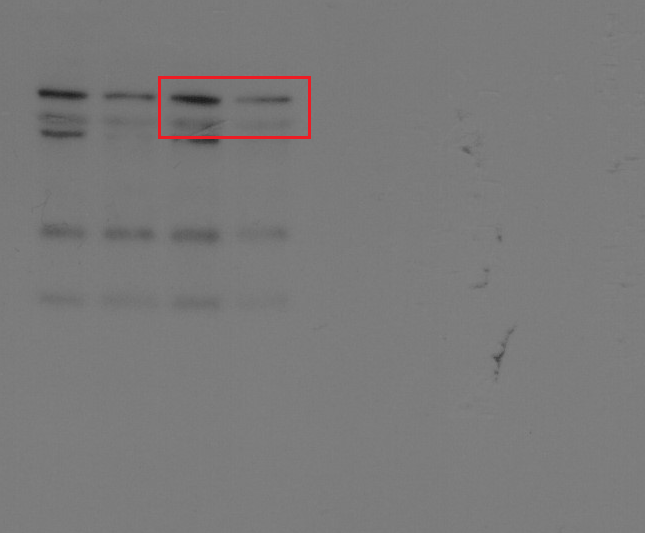

Supplement: Supplementary file 8 — Source Data [file 41467_2023_39787_MOESM8_ESM.zip › gels_blots/Figure 5/Fig. 5j-hnRNP R.tif]

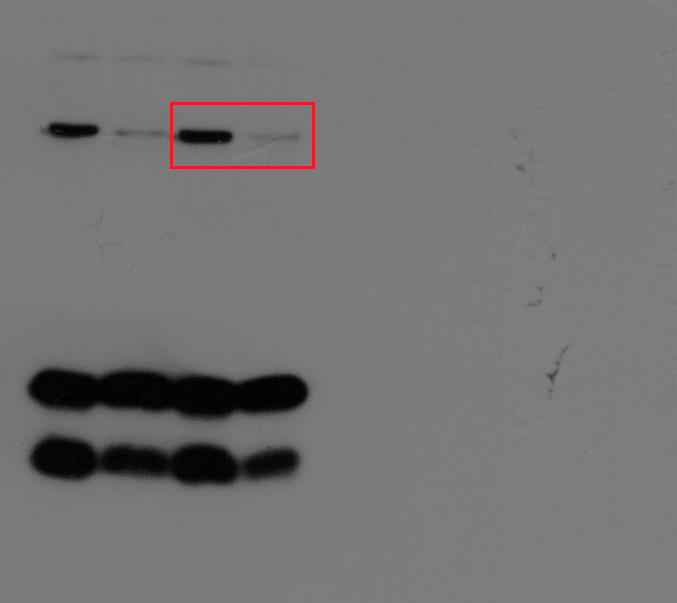

Supplement: Supplementary file 8 — Source Data [file 41467_2023_39787_MOESM8_ESM.zip › gels_blots/Figure 5/Fig. 5j-Ptbp2.tif]

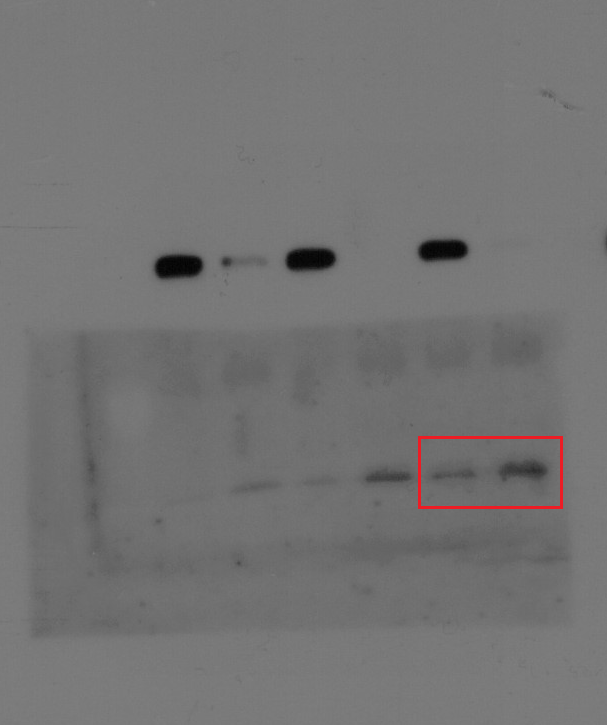

Supplement: Supplementary file 8 — Source Data [file 41467_2023_39787_MOESM8_ESM.zip › gels_blots/Figure 6/Fig. 6d-EGFP.tif]

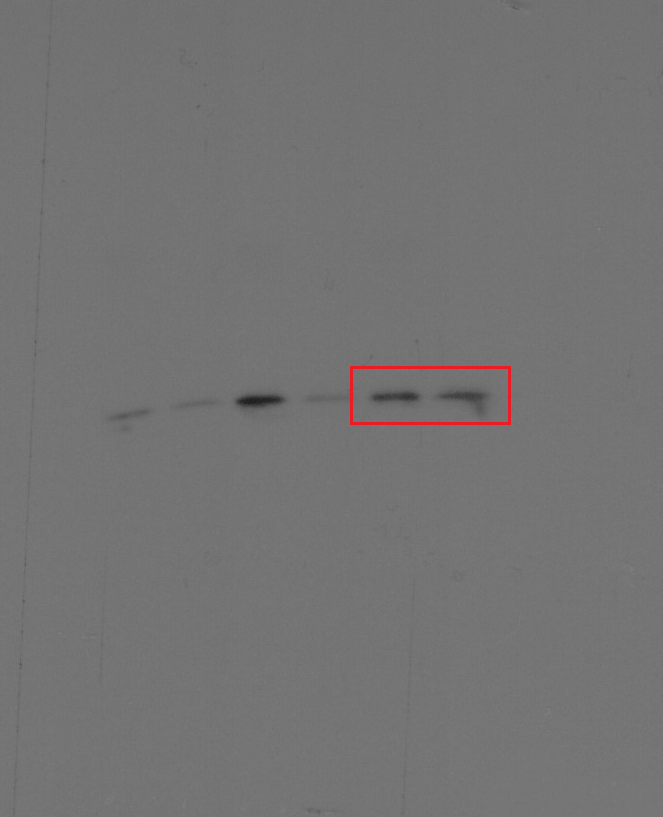

Supplement: Supplementary file 8 — Source Data [file 41467_2023_39787_MOESM8_ESM.zip › gels_blots/Figure 6/Fig. 6d-Histone H3.tif]

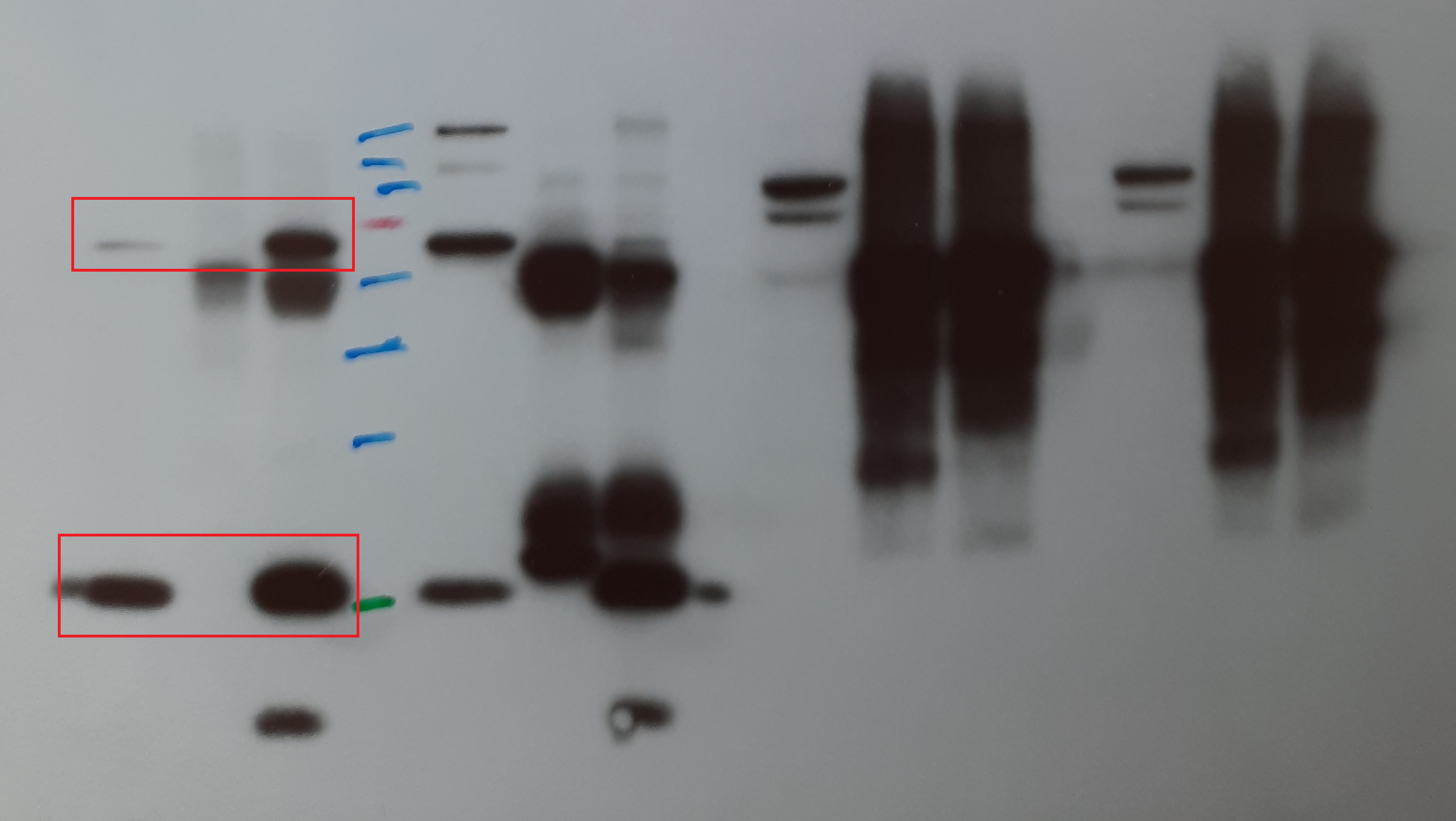

Supplement: Supplementary file 8 — Source Data [file 41467_2023_39787_MOESM8_ESM.zip › gels_blots/Figure 6/Fig. 6f-Ptbp2-Rps5.tif]

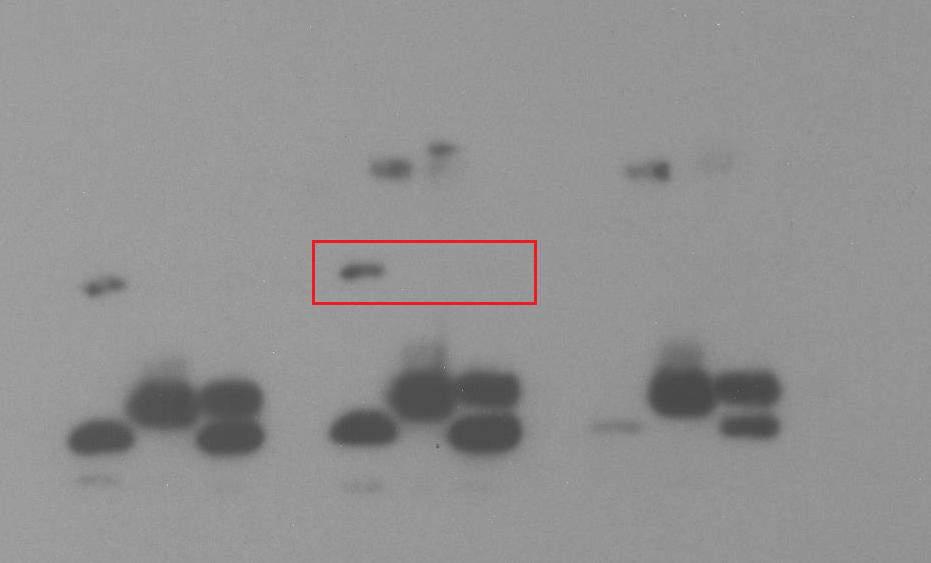

Supplement: Supplementary file 8 — Source Data [file 41467_2023_39787_MOESM8_ESM.zip › gels_blots/Figure 6/Fig. 6g-Gapdh.tif]

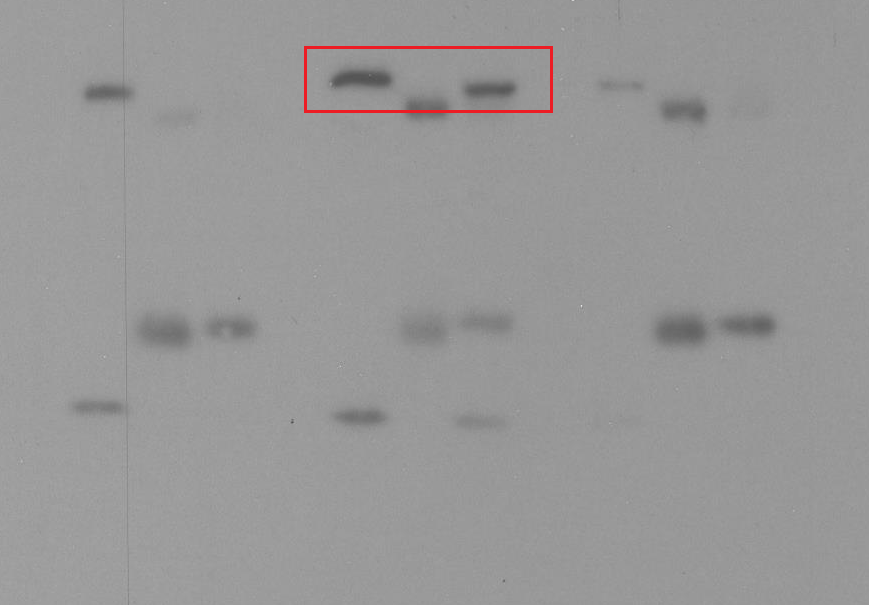

Supplement: Supplementary file 8 — Source Data [file 41467_2023_39787_MOESM8_ESM.zip › gels_blots/Figure 6/Fig. 6g-Ptbp2.tif]

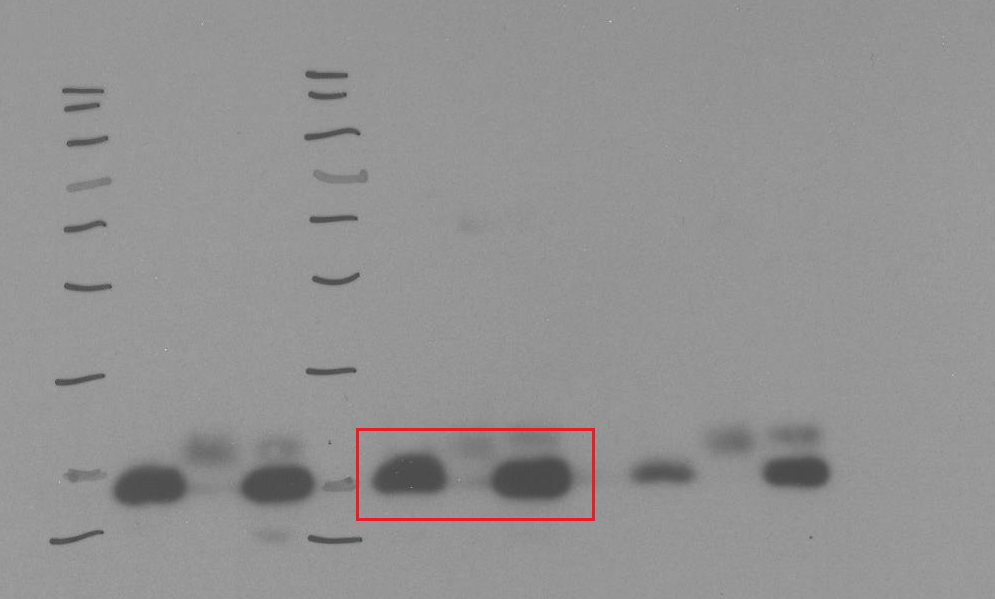

Supplement: Supplementary file 8 — Source Data [file 41467_2023_39787_MOESM8_ESM.zip › gels_blots/Figure 6/Fig. 6g-Rps5.tif]

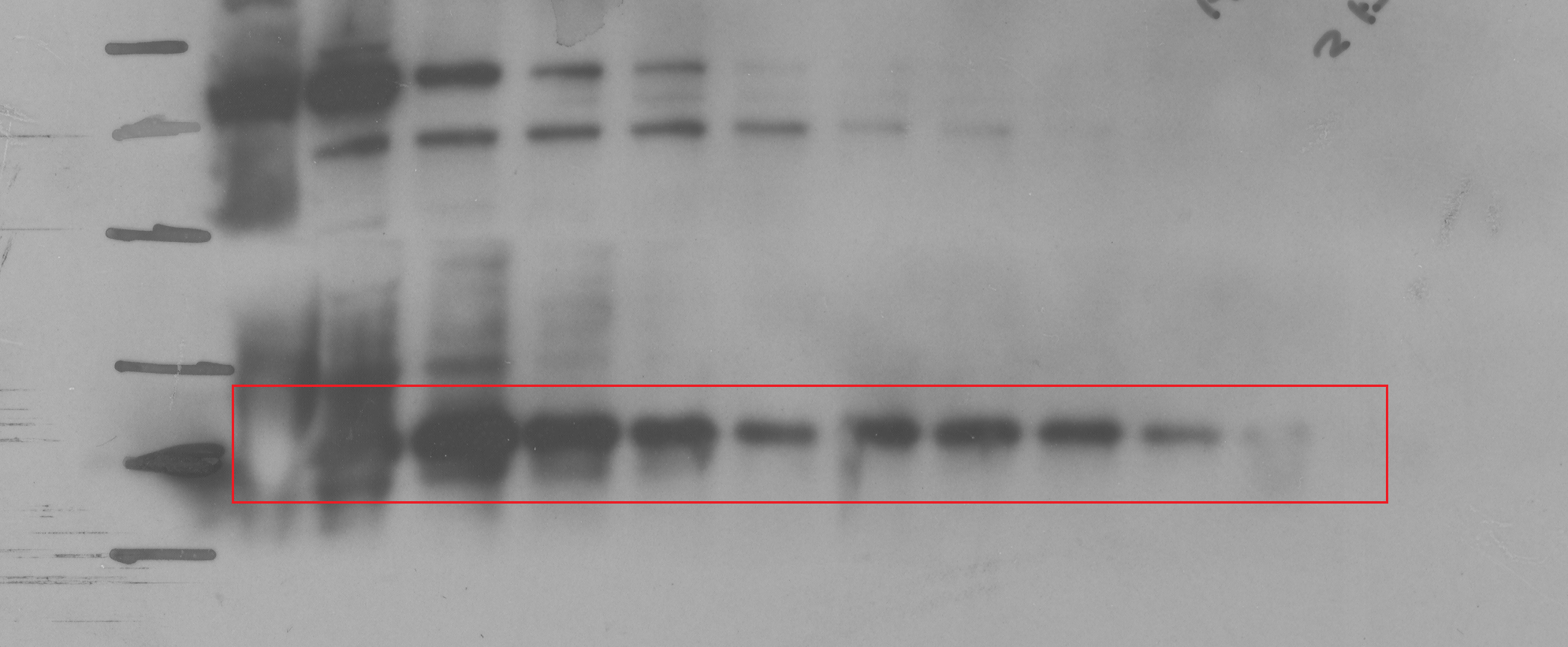

Supplement: Supplementary file 8 — Source Data [file 41467_2023_39787_MOESM8_ESM.zip › gels_blots/Figure 6/Fig. 6h-eIF2a.tif]

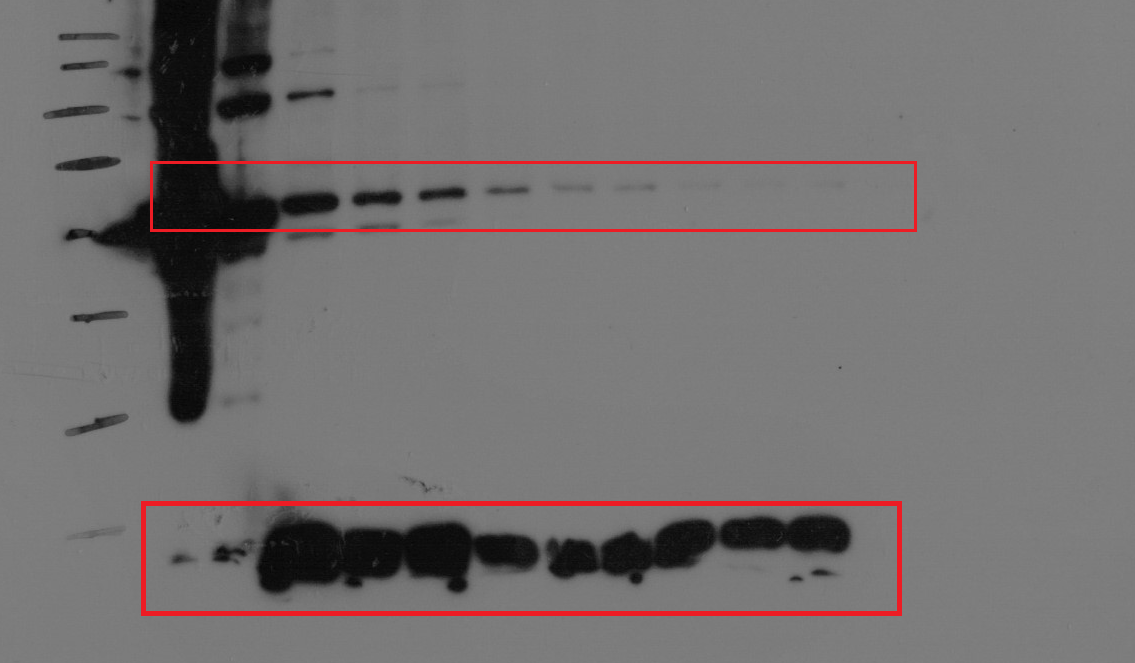

Supplement: Supplementary file 8 — Source Data [file 41467_2023_39787_MOESM8_ESM.zip › gels_blots/Figure 6/Fig. 6h-Ptbp2-Rps5.tif]

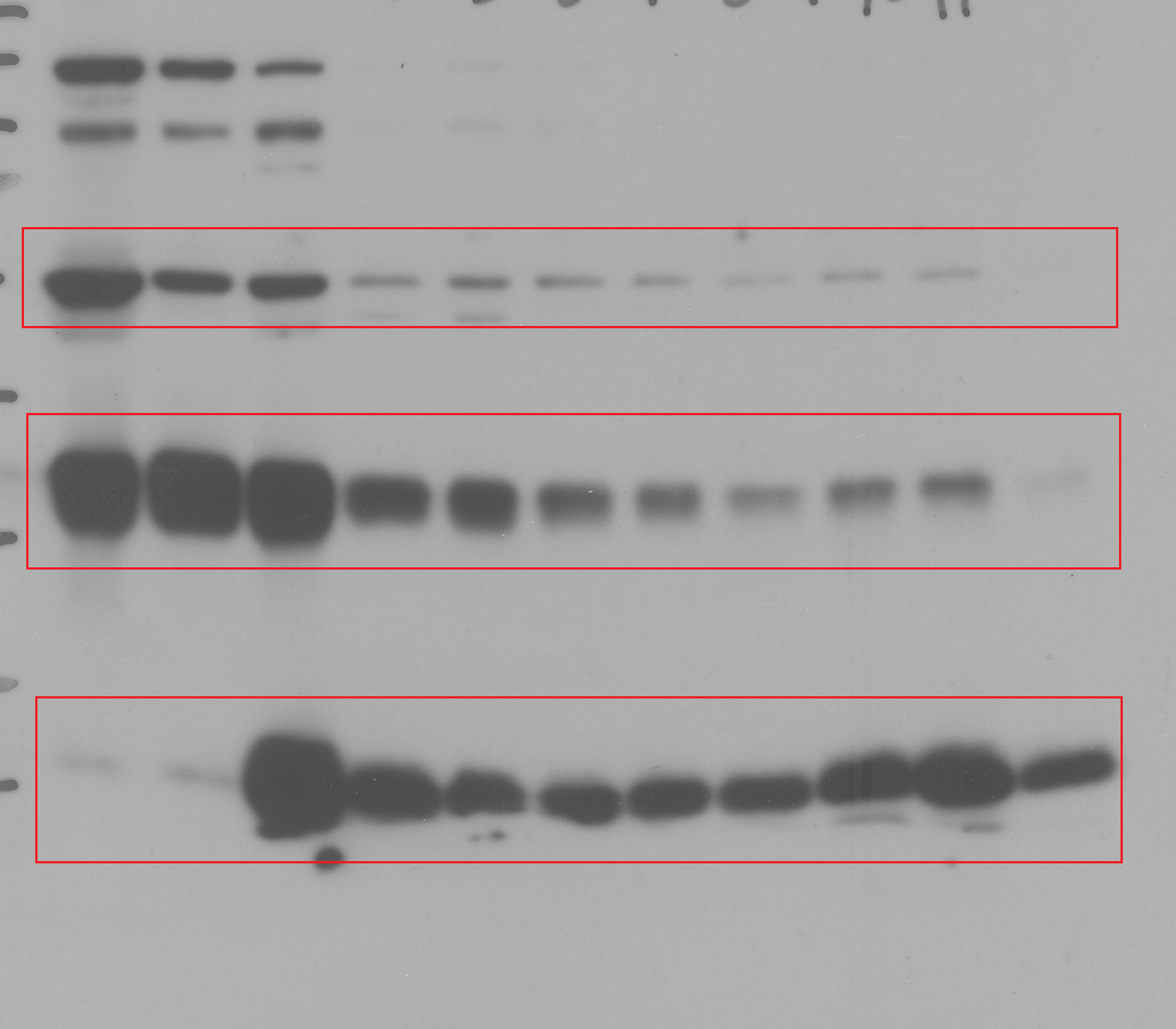

Supplement: Supplementary file 8 — Source Data [file 41467_2023_39787_MOESM8_ESM.zip › gels_blots/Figure 6/Fig. 6j-Ctrl.tif]

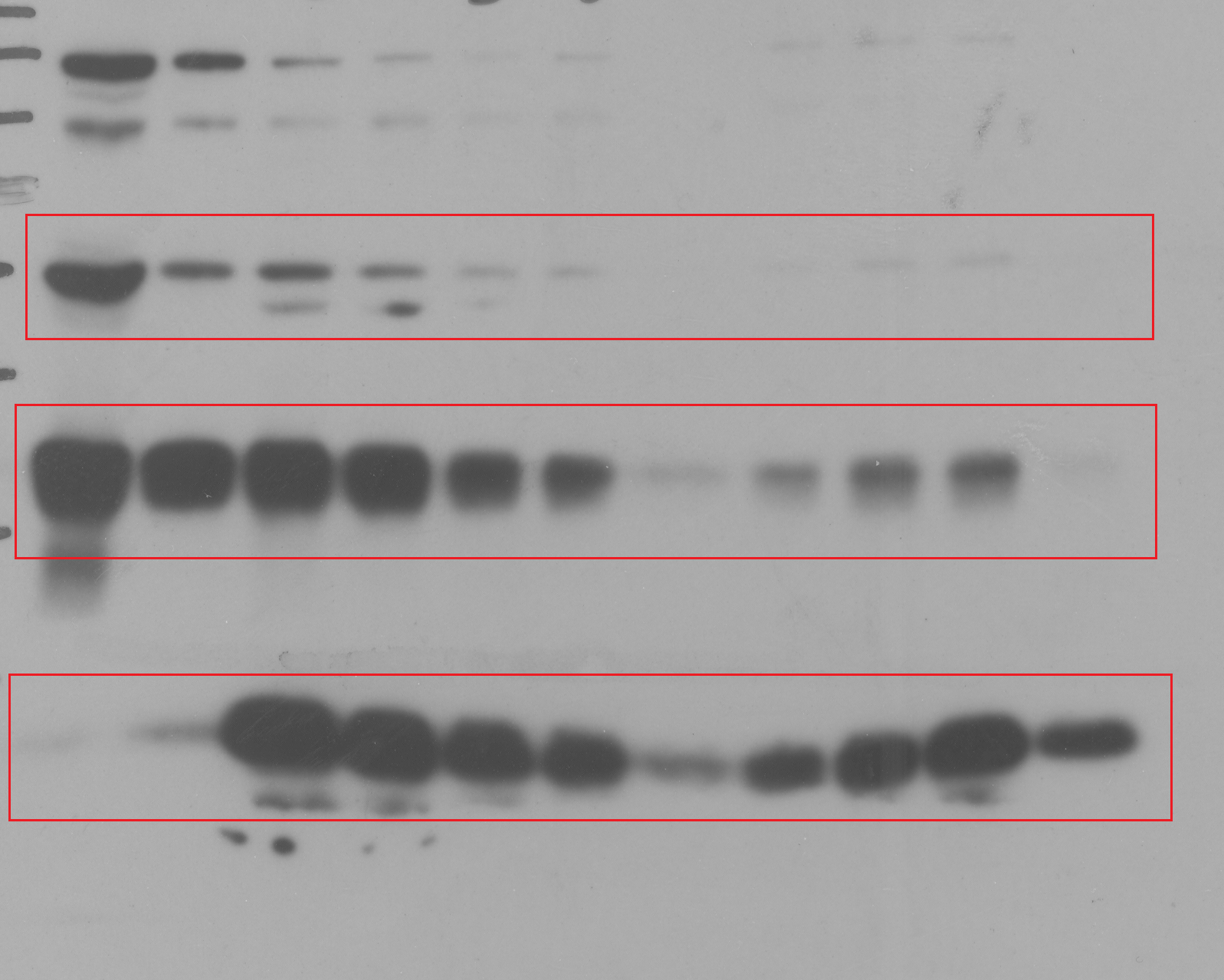

Supplement: Supplementary file 8 — Source Data [file 41467_2023_39787_MOESM8_ESM.zip › gels_blots/Figure 6/Fig. 6j-sh1Ptbp2.tif]

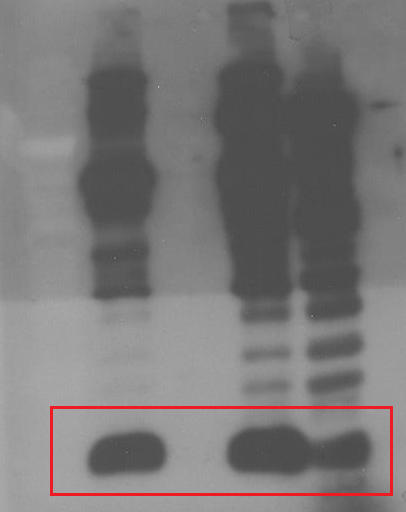

Supplement: Supplementary file 8 — Source Data [file 41467_2023_39787_MOESM8_ESM.zip › gels_blots/Figure 7/Fig. 7a-eIF5A.tif]

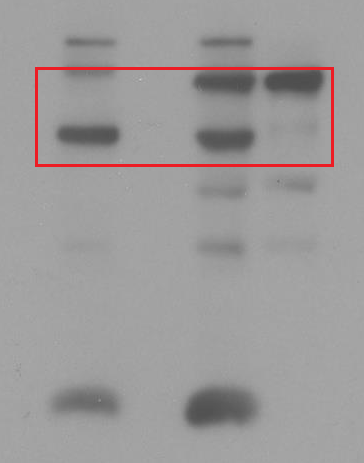

Supplement: Supplementary file 8 — Source Data [file 41467_2023_39787_MOESM8_ESM.zip › gels_blots/Figure 7/Fig. 7a-Ptbp2 and Ptbp2-EGFP.tif]

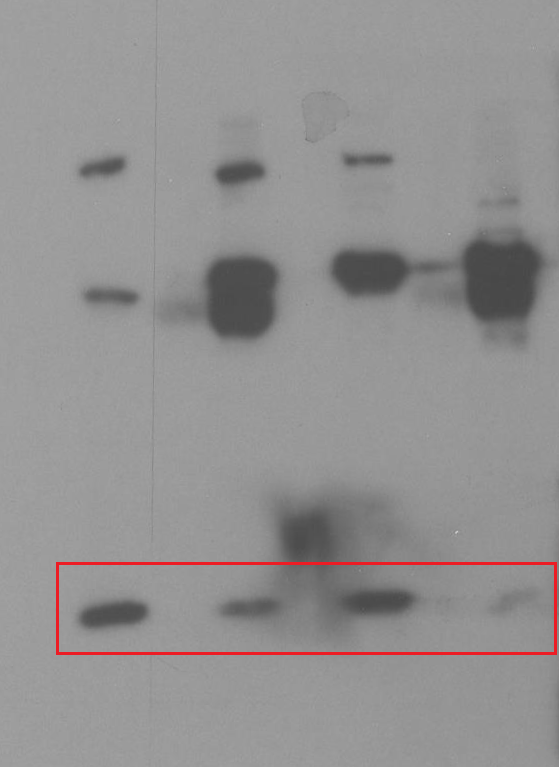

Supplement: Supplementary file 8 — Source Data [file 41467_2023_39787_MOESM8_ESM.zip › gels_blots/Figure 7/Fig. 7c-eIF5A.tif]

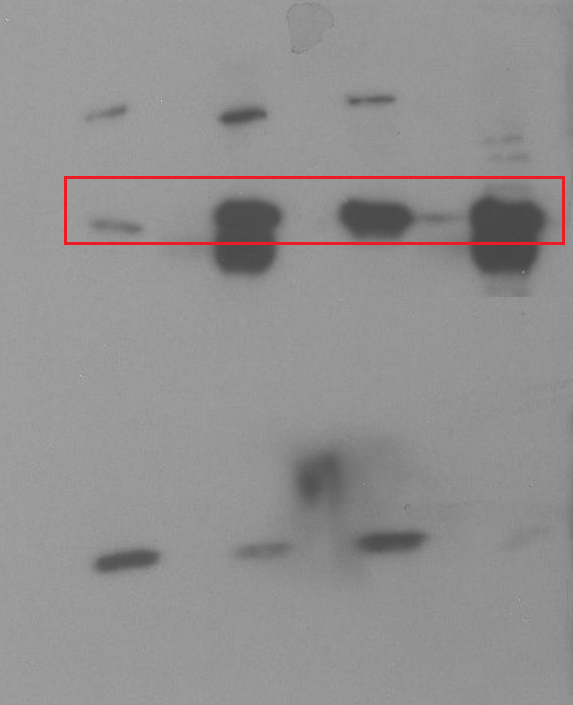

Supplement: Supplementary file 8 — Source Data [file 41467_2023_39787_MOESM8_ESM.zip › gels_blots/Figure 7/Fig. 7c-Ptbp2.tif]

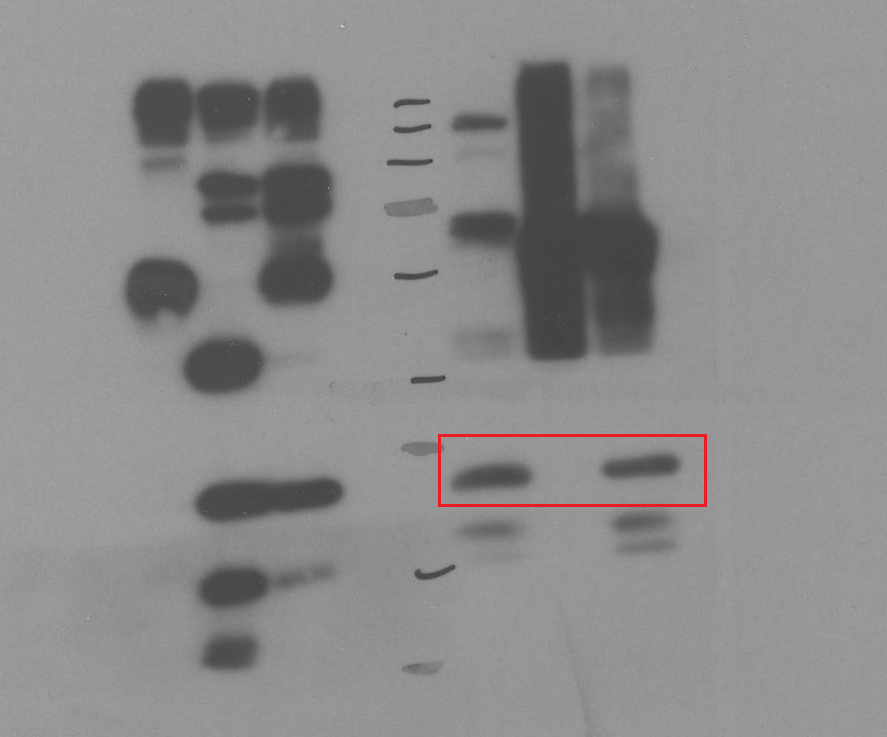

Supplement: Supplementary file 8 — Source Data [file 41467_2023_39787_MOESM8_ESM.zip › gels_blots/Figure 7/Fig. 7d-eIF5A.tif]

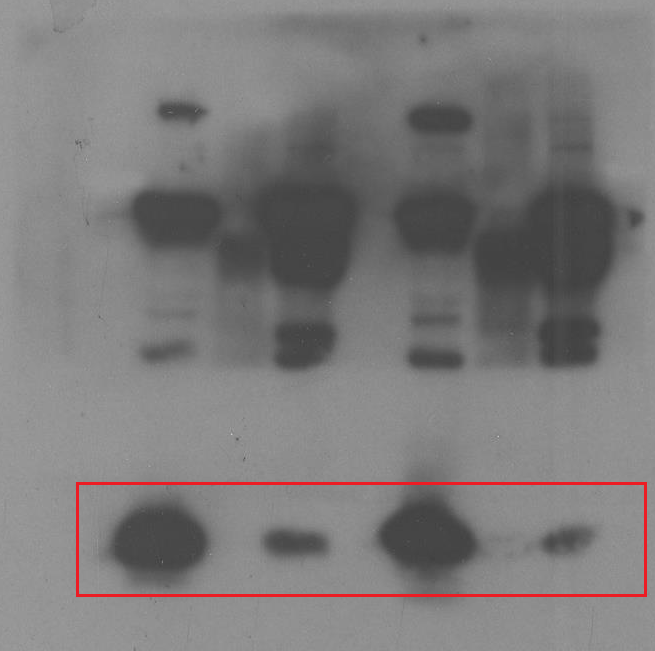

Supplement: Supplementary file 8 — Source Data [file 41467_2023_39787_MOESM8_ESM.zip › gels_blots/Figure 7/Fig. 7h-eIF5A.tif]

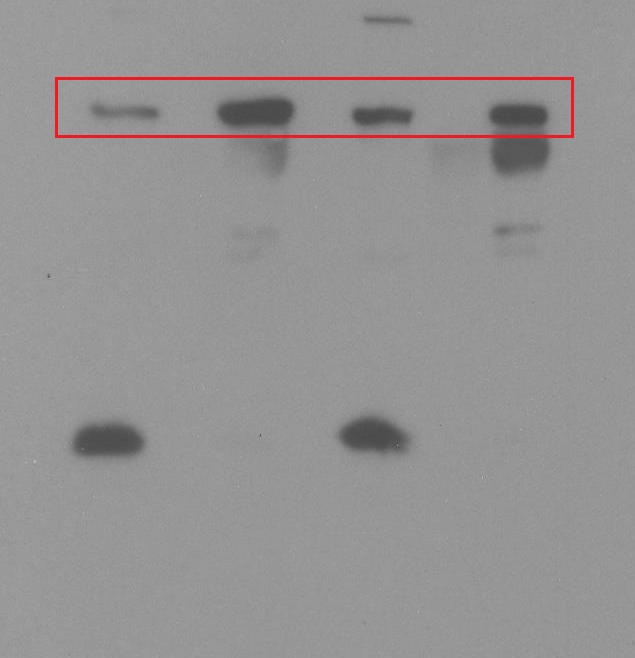

Supplement: Supplementary file 8 — Source Data [file 41467_2023_39787_MOESM8_ESM.zip › gels_blots/Figure 7/Fig. 7h-Ptbp2.tif]

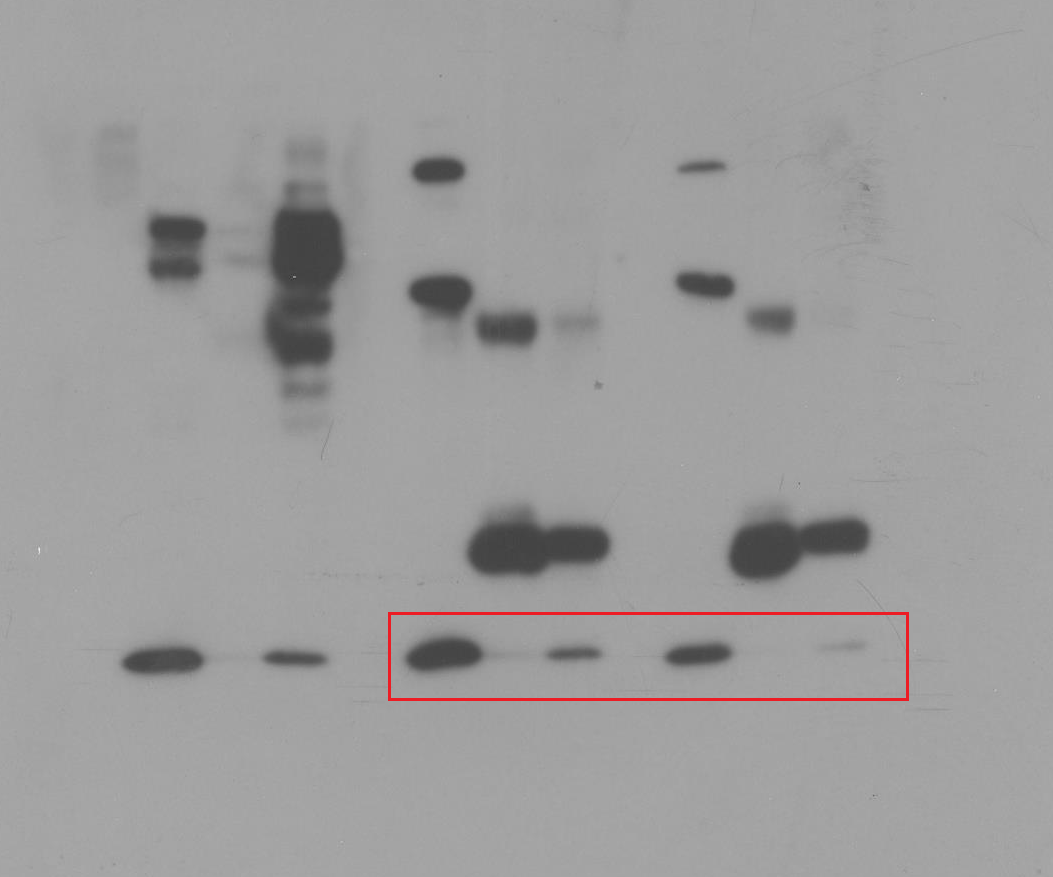

Supplement: Supplementary file 8 — Source Data [file 41467_2023_39787_MOESM8_ESM.zip › gels_blots/Figure 7/Fig. 7i-eIF5A.tif]

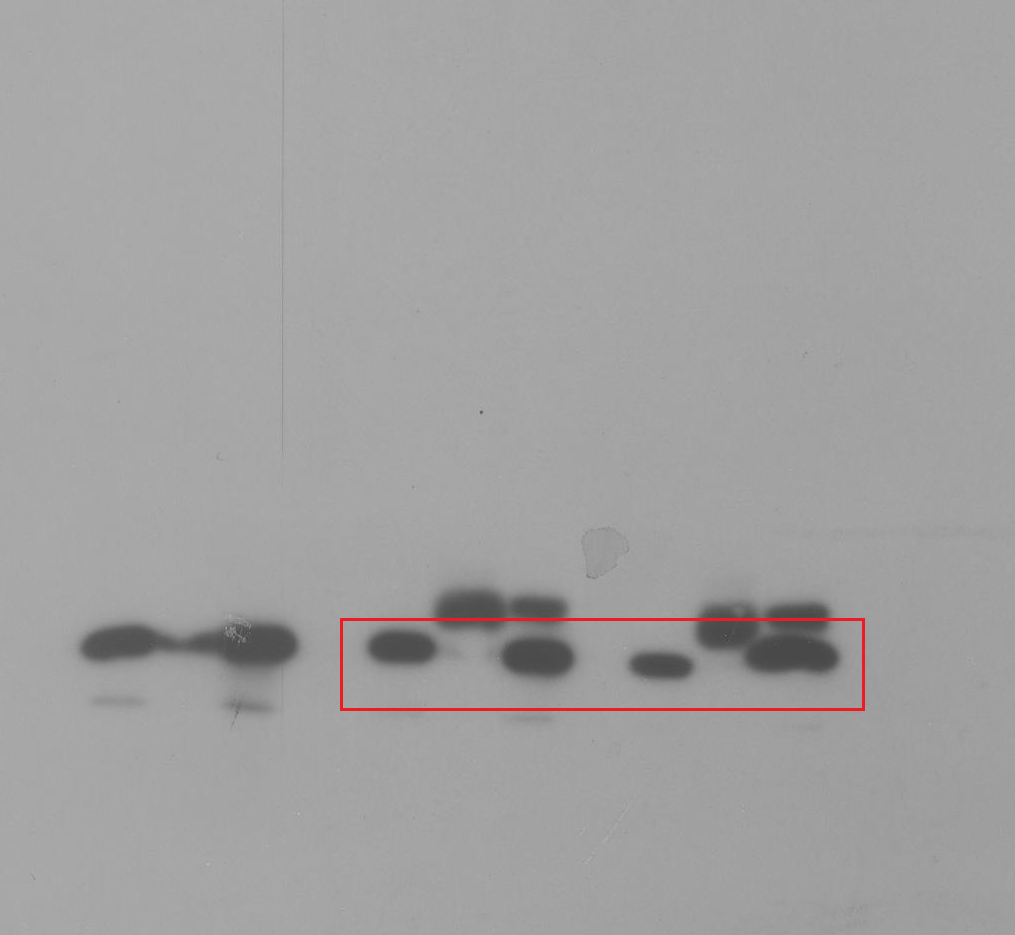

Supplement: Supplementary file 8 — Source Data [file 41467_2023_39787_MOESM8_ESM.zip › gels_blots/Figure 7/Fig. 7i-Rps5.tif]

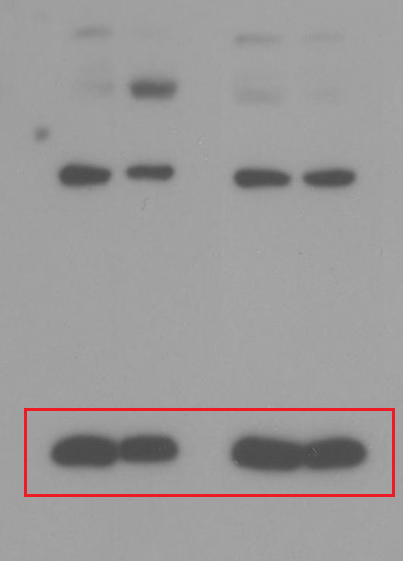

Supplement: Supplementary file 8 — Source Data [file 41467_2023_39787_MOESM8_ESM.zip › gels_blots/Figure 7/Fig. 7k-Histone H3.tif]

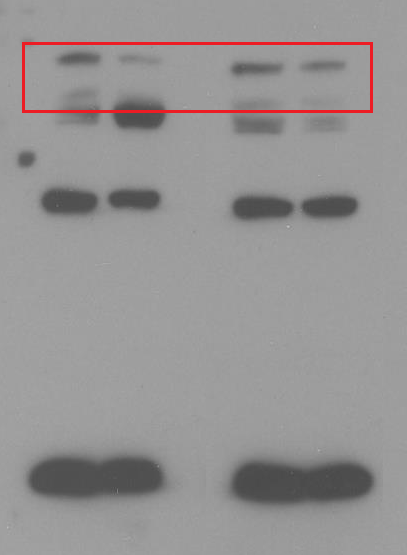

Supplement: Supplementary file 8 — Source Data [file 41467_2023_39787_MOESM8_ESM.zip › gels_blots/Figure 7/Fig. 7k-hnRNP R.tif]

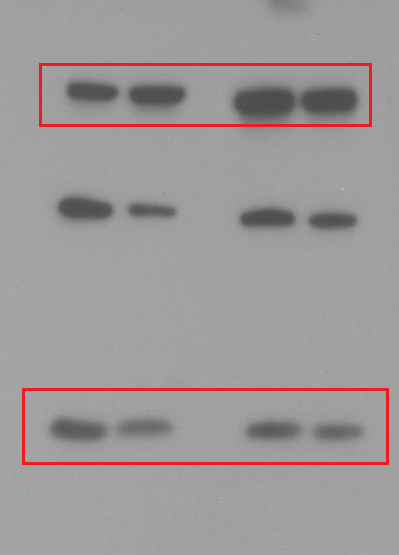

Supplement: Supplementary file 8 — Source Data [file 41467_2023_39787_MOESM8_ESM.zip › gels_blots/Figure 7/Fig. 7k-Ptbp2-eIF5A.tif]

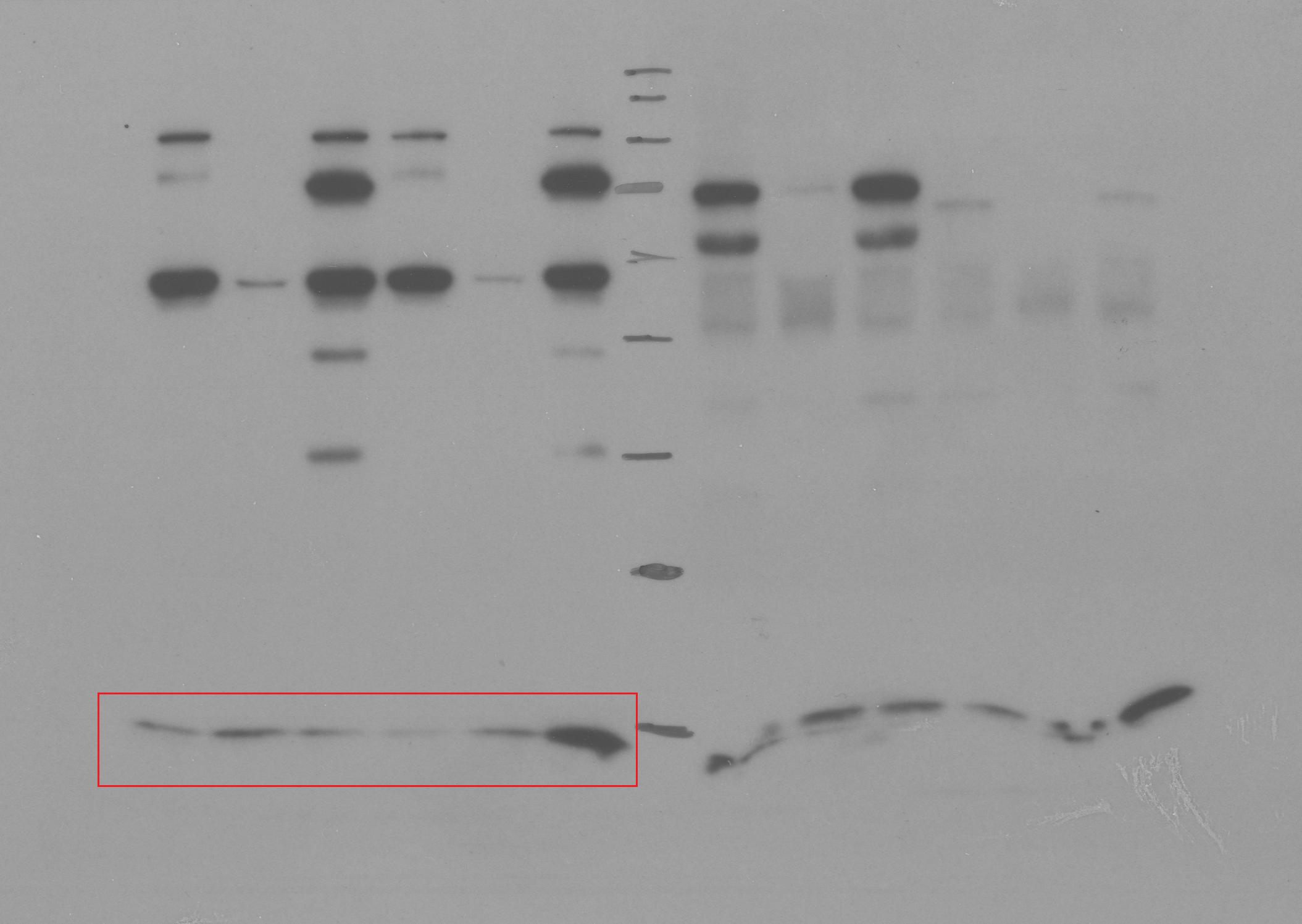

Supplement: Supplementary file 8 — Source Data [file 41467_2023_39787_MOESM8_ESM.zip › gels_blots/Figure 8/Fig. 8h- Histone H3.tif]

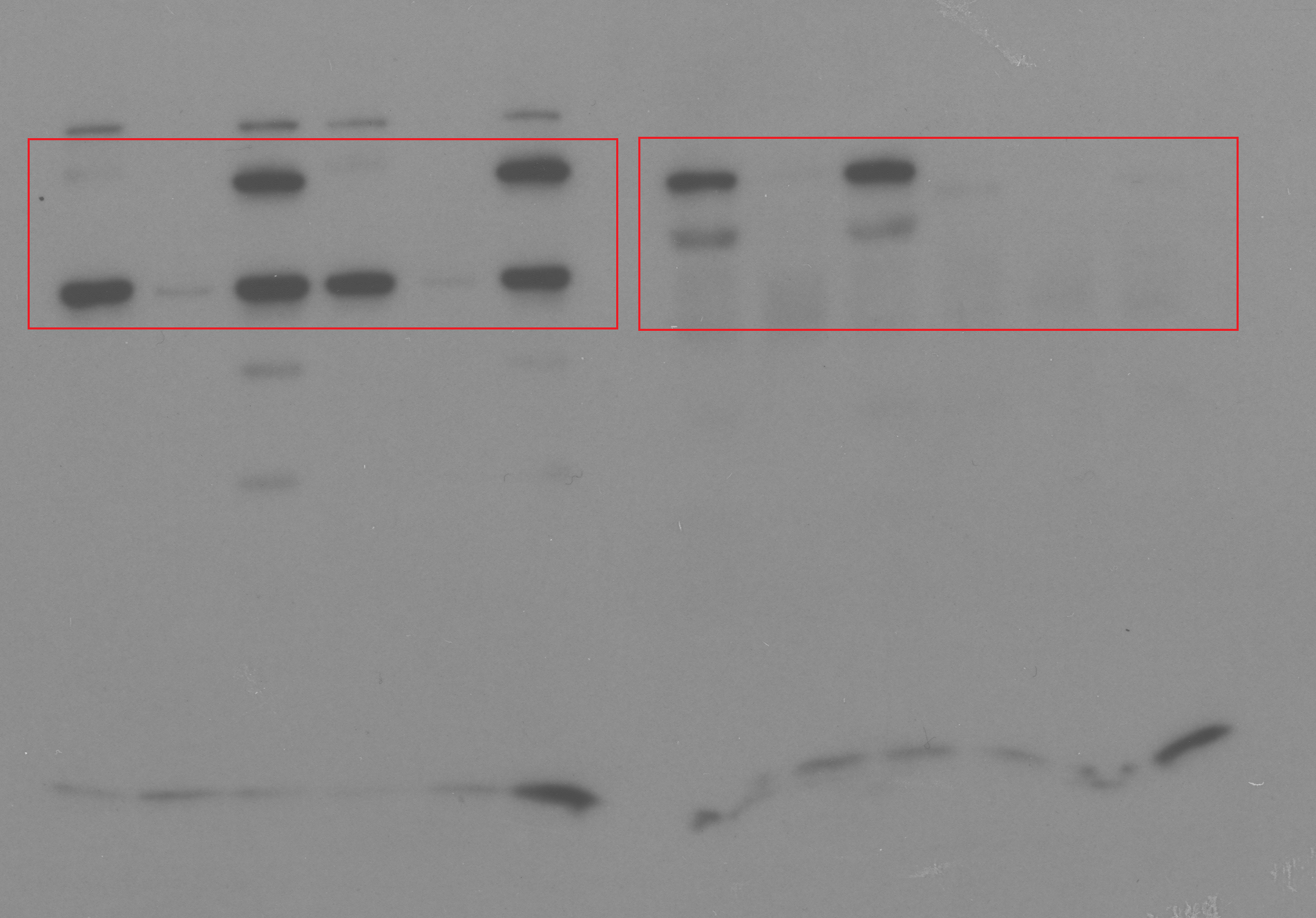

Supplement: Supplementary file 8 — Source Data [file 41467_2023_39787_MOESM8_ESM.zip › gels_blots/Figure 8/Fig. 8h- Ptbp2-hnRNP R.tif]

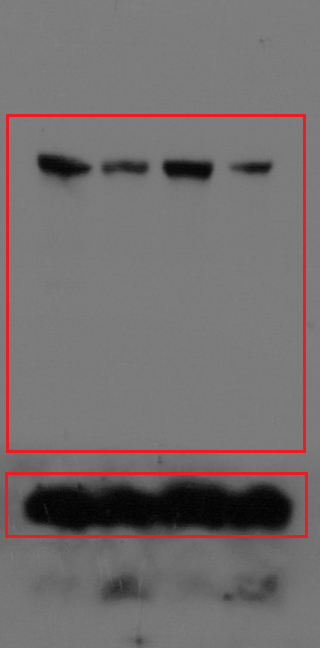

Supplement: Supplementary file 8 — Source Data [file 41467_2023_39787_MOESM8_ESM.zip › gels_blots/Supplementary Figure 1/Fig. S1a-Long exposure.tif]

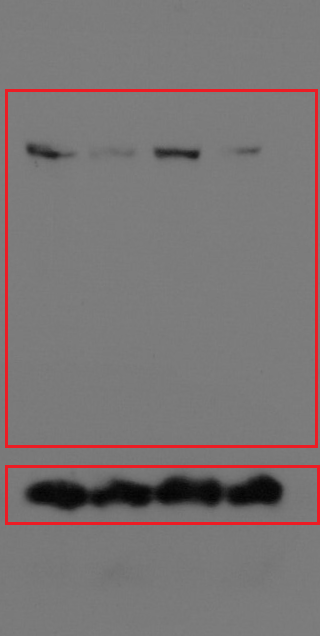

Supplement: Supplementary file 8 — Source Data [file 41467_2023_39787_MOESM8_ESM.zip › gels_blots/Supplementary Figure 1/Fig. S1a-Short exposure.tif]

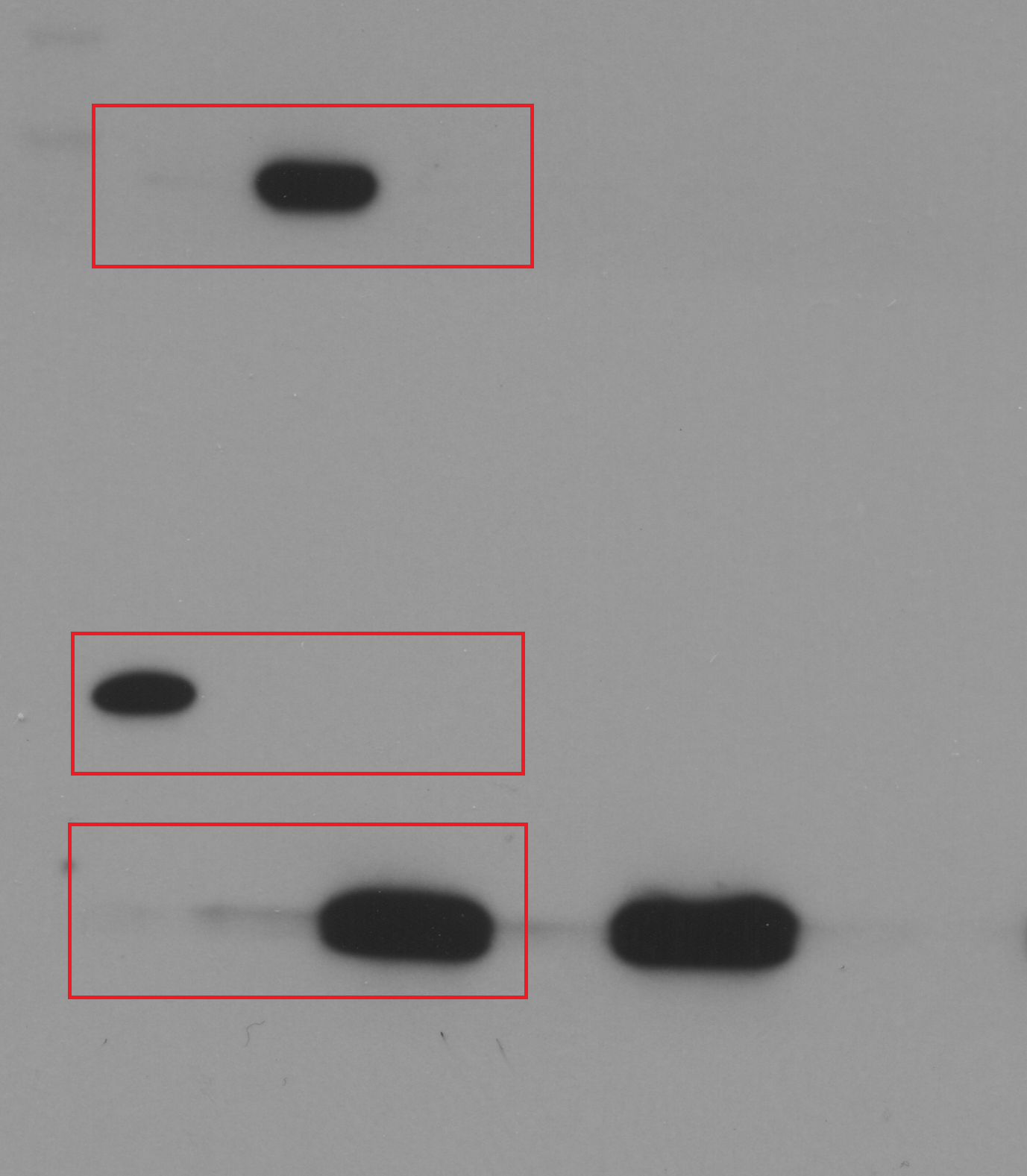

Supplement: Supplementary file 8 — Source Data [file 41467_2023_39787_MOESM8_ESM.zip › gels_blots/Supplementary Figure 1/Fig. S1c-Calnexin-Gapdh-Histone H3.tif]

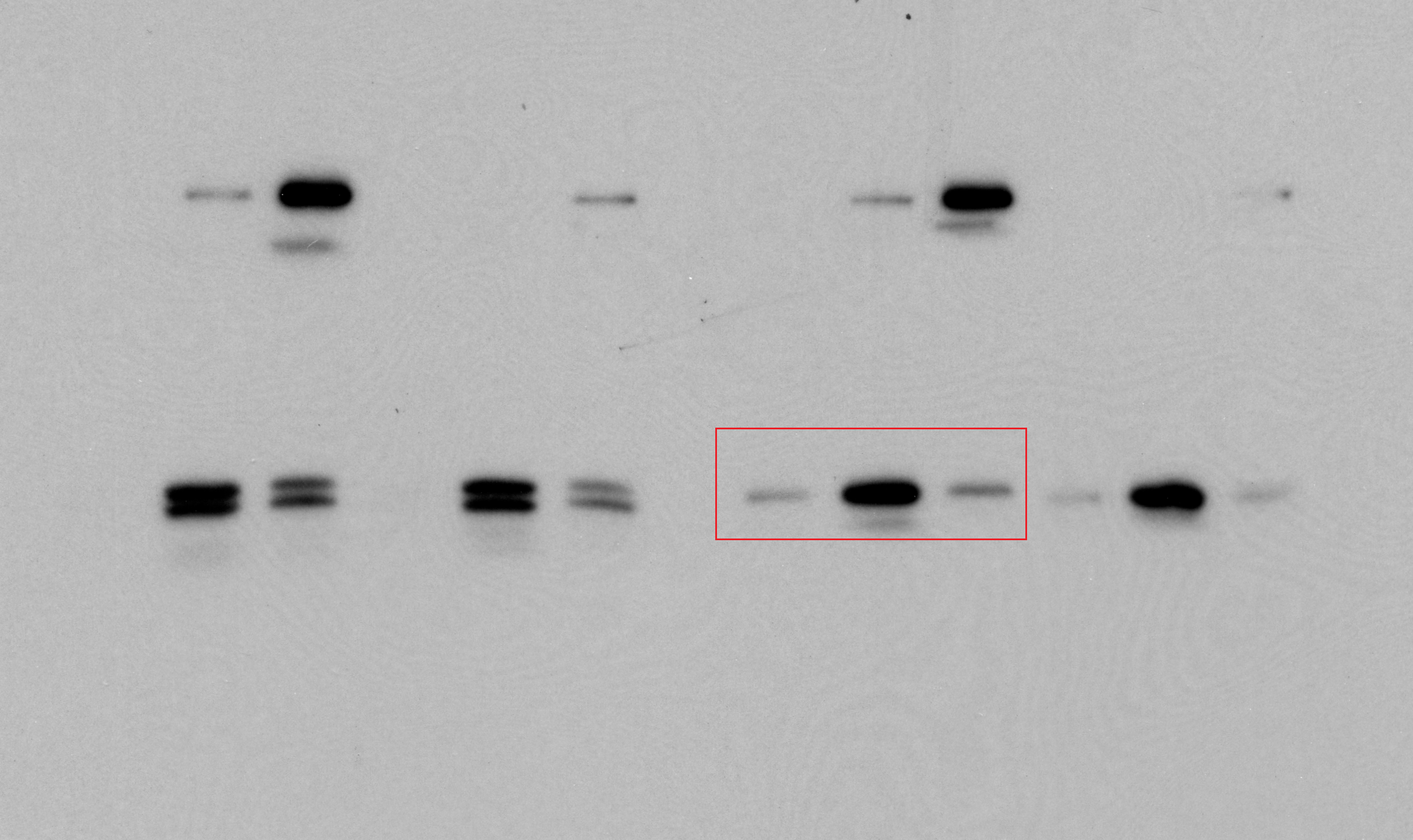

Supplement: Supplementary file 8 — Source Data [file 41467_2023_39787_MOESM8_ESM.zip › gels_blots/Supplementary Figure 1/Fig. S1c-Ptbp2.tif]

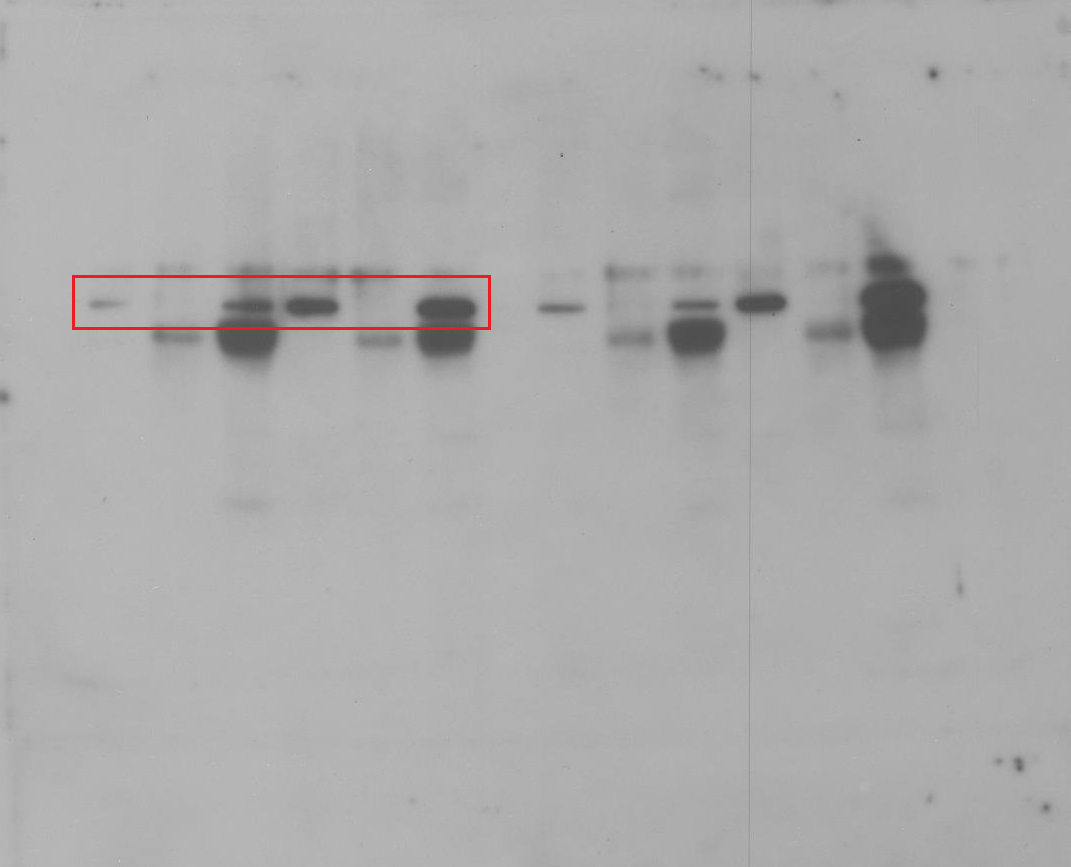

Supplement: Supplementary file 8 — Source Data [file 41467_2023_39787_MOESM8_ESM.zip › gels_blots/Supplementary Figure 3/Fig. S3c-Ptbp2.tif]

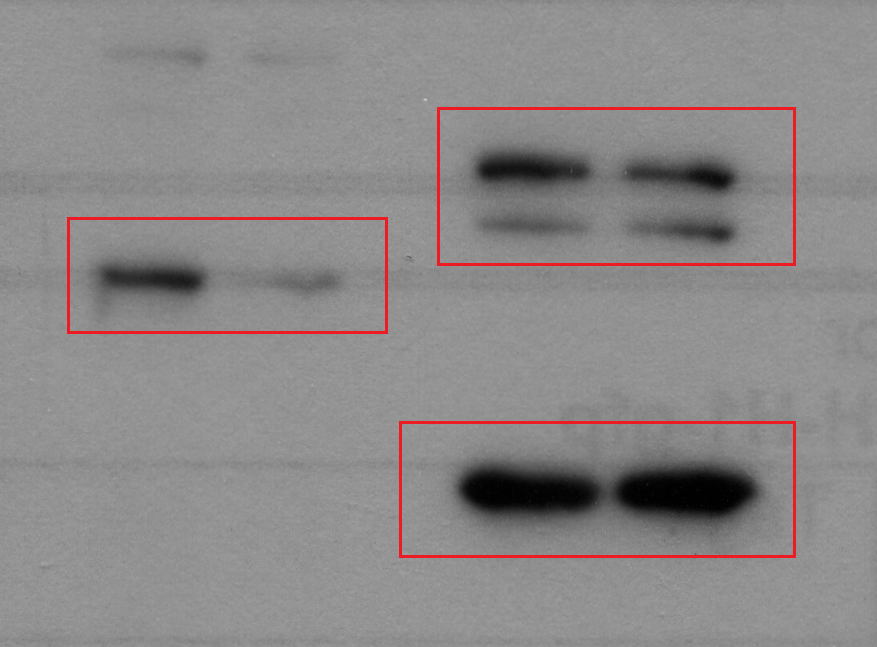

Supplement: Supplementary file 8 — Source Data [file 41467_2023_39787_MOESM8_ESM.zip › gels_blots/Supplementary Figure 4/Fig. S4d-Ptbp2-hnRNP R-Gapdh.png]

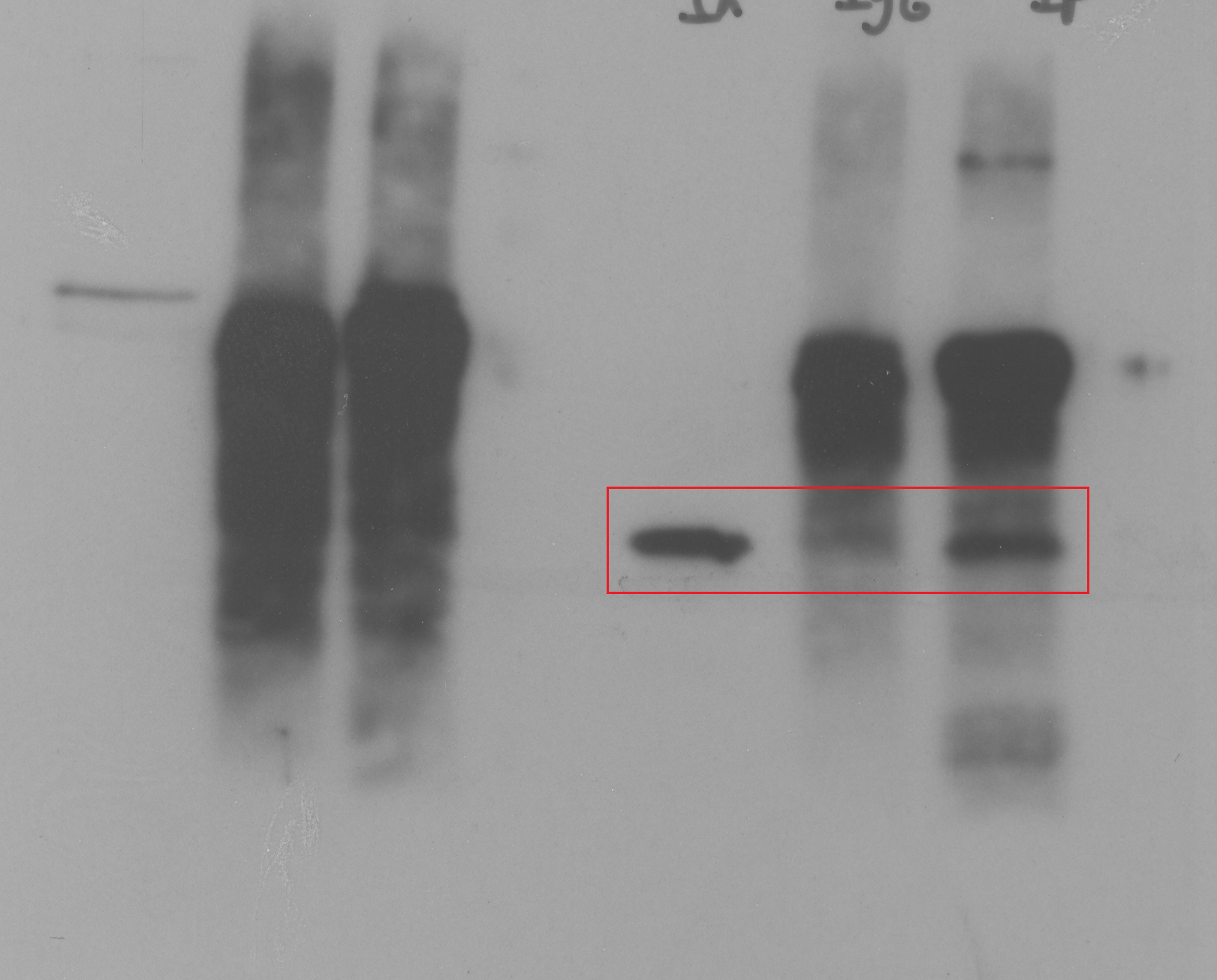

Supplement: Supplementary file 8 — Source Data [file 41467_2023_39787_MOESM8_ESM.zip › gels_blots/Supplementary Figure 5/Fig. S5a. eIF2a.tif]

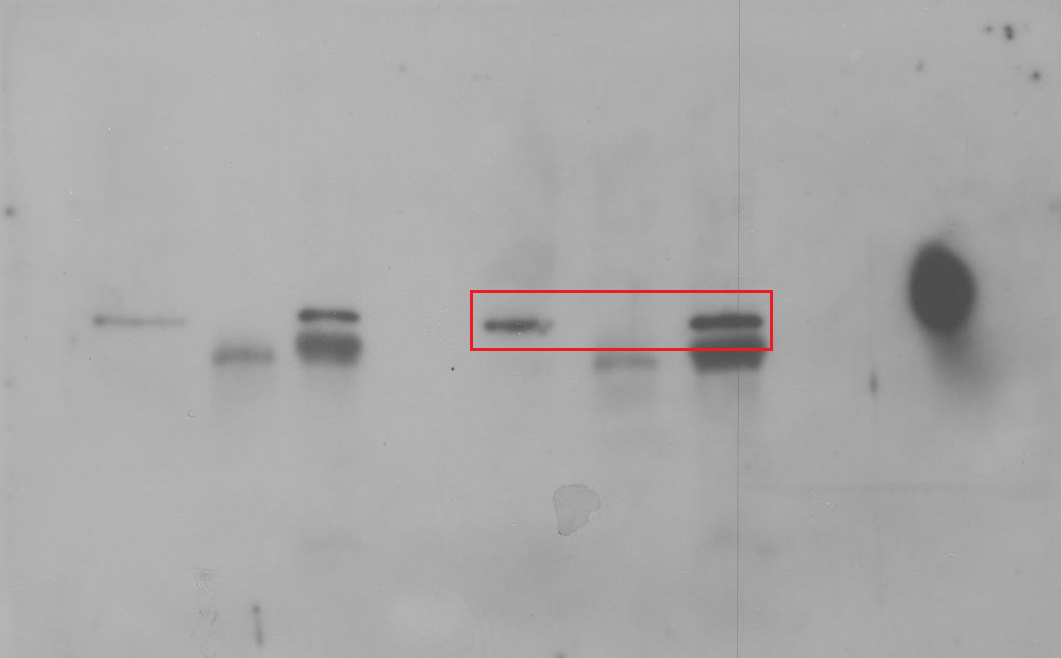

Supplement: Supplementary file 8 — Source Data [file 41467_2023_39787_MOESM8_ESM.zip › gels_blots/Supplementary Figure 5/Fig. S5a. Ptbp2.tif]

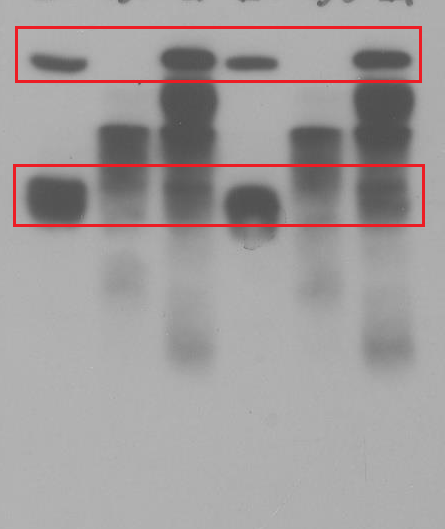

Supplement: Supplementary file 8 — Source Data [file 41467_2023_39787_MOESM8_ESM.zip › gels_blots/Supplementary Figure 5/Fig. S5b-Ptbp2-eIF2a.tif]

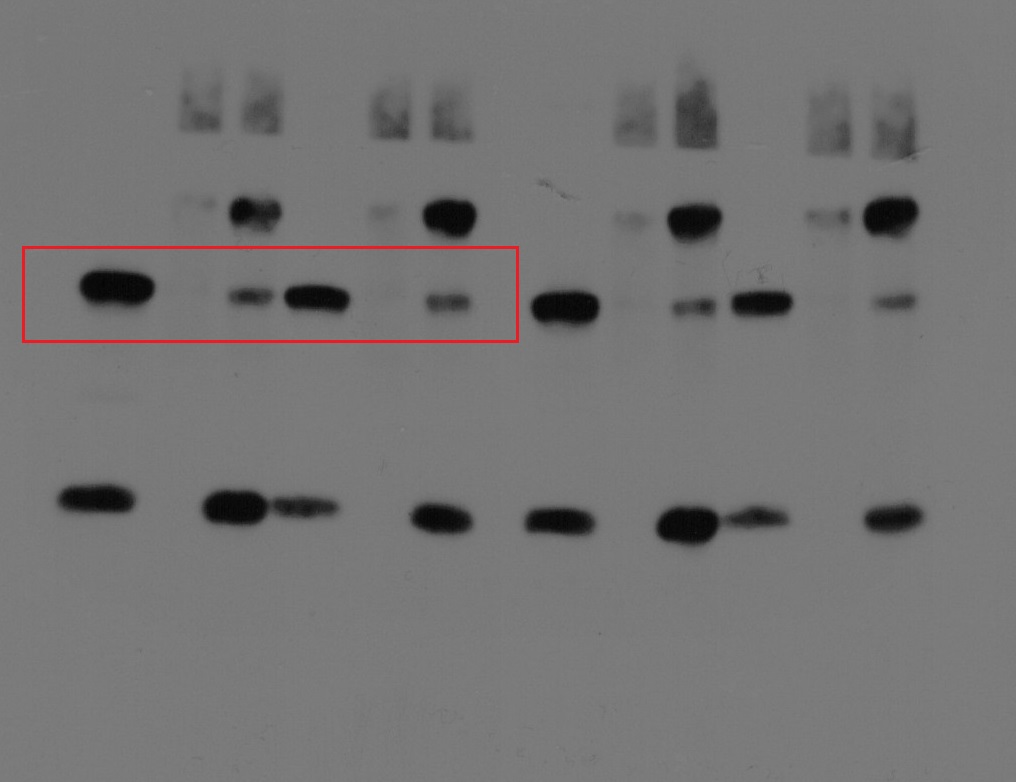

Supplement: Supplementary file 8 — Source Data [file 41467_2023_39787_MOESM8_ESM.zip › gels_blots/Supplementary Figure 5/Fig. S5c. eIF2a.tif]

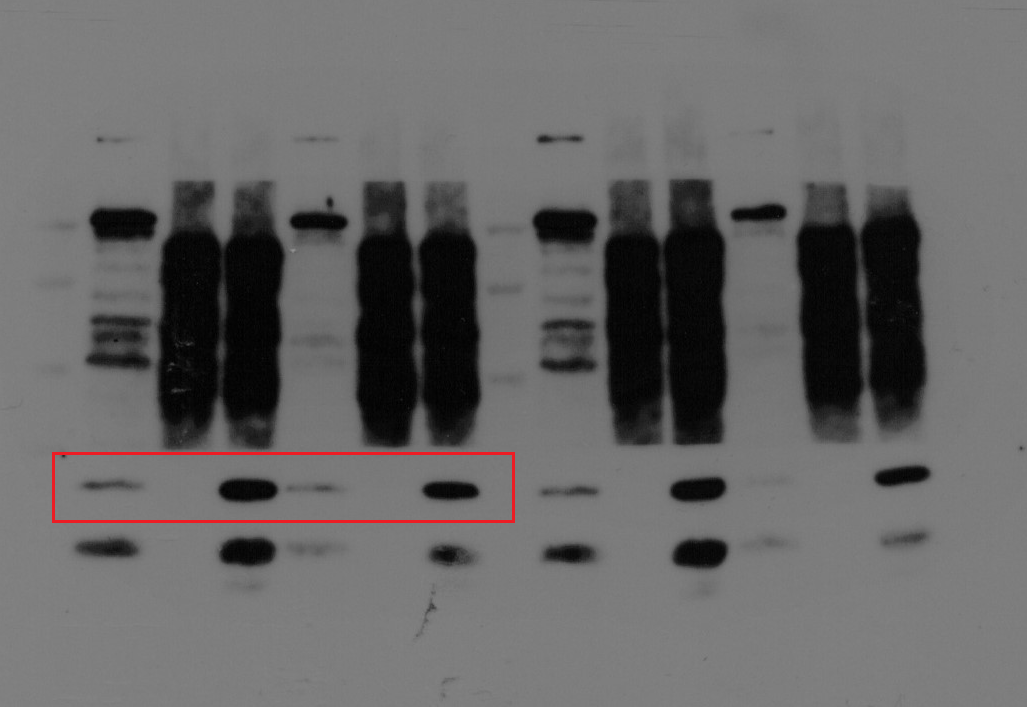

Supplement: Supplementary file 8 — Source Data [file 41467_2023_39787_MOESM8_ESM.zip › gels_blots/Supplementary Figure 5/Fig. S5c. Rps5.tif]

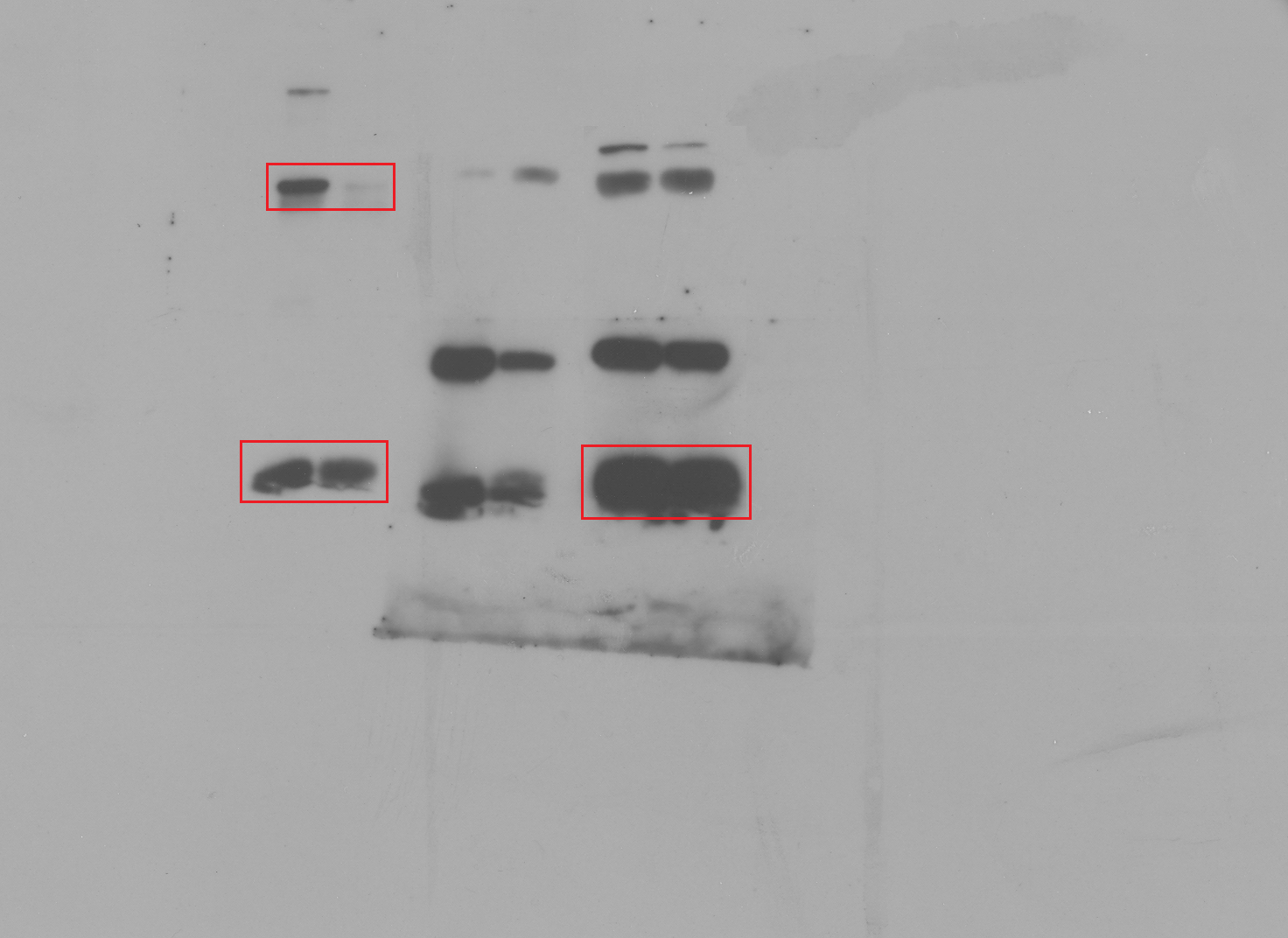

Supplement: Supplementary file 8 — Source Data [file 41467_2023_39787_MOESM8_ESM.zip › gels_blots/Supplementary Figure 5/Fig. S5e. Ptbp2-eIF5A-Histone H3.tif]
